# Public knowledge, beliefs and behaviours related to antimicrobial resistance and antibiotics: a systematic review of studies in high-income countries

**DOI:** 10.1093/jacamr/dlag213

**Published:** 2026-09-30

**Authors:** Caoimhe Shields, Tracy Epton, Emma Berry, Laura J Sahm, Aoife Fleming, Ilias Lambrou, Rebecca Feeney, Najam us Sahar, Mitch Dobbs, Chikondi C Kandulu, Ellen Melia, Gillian W Shorter

**Affiliations:** School of Psychology, Queen’s University Belfast, University Road, Belfast BT7 1NN, Northern Ireland; Manchester Centre for Health Psychology, University of Manchester, Manchester, UK; School of Psychology, Queen’s University Belfast, University Road, Belfast BT7 1NN, Northern Ireland; Pharmaceutical Care Research Group, School of Pharmacy, University College Cork, Cork, Ireland; Pharmacy Department, Mercy University Hospital, Cork, Ireland; Pharmaceutical Care Research Group, School of Pharmacy, University College Cork, Cork, Ireland; Pharmacy Department, Mercy University Hospital, Cork, Ireland; School of Psychology, Queen’s University Belfast, University Road, Belfast BT7 1NN, Northern Ireland; School of Psychology, Queen’s University Belfast, University Road, Belfast BT7 1NN, Northern Ireland; School of Psychology, Queen’s University Belfast, University Road, Belfast BT7 1NN, Northern Ireland; Department of Psychology, Northeastern University, Boston, MA, USA; Pharmaceutical Care Research Group, School of Pharmacy, University College Cork, Cork, Ireland; Pharmaceutical Care Research Group, School of Pharmacy, University College Cork, Cork, Ireland; School of Psychology, Queen’s University Belfast, University Road, Belfast BT7 1NN, Northern Ireland; School of Nursing and Midwifery, Trinity College Dublin, The University of Dublin, Dublin, Ireland; TreAdd Research Group on Treatment and Addictions, Tampere University, Tampere, Finland

## Abstract

**Background:**

Antimicrobial resistance (AMR) is a growing public health concern, influenced by public behaviours. This study aimed to systematically review and synthesize qualitative and quantitative studies on public knowledge, beliefs and behaviours related to antibiotics and AMR, and explore gender differences, in high-income countries.

**Materials and methods:**

Eleven databases including Web of Science Core Collection, Medline and PsycINFO were searched for primary studies published between August 2014 and 12 June 2026. This article reports the high-income country subset of a larger global review; other results will be reported separately. We carried out a descriptive, non-meta-analytic synthesis at study- and country-level by calculating the mean, SD, median and range of the proportion of participants whose data demonstrated incorrect knowledge, misconceptions, and positive or negative behaviours. Gender differences were synthesized narratively. Qualitative data were synthesized under each outcome using deductive thematic analysis.

**Results:**

Of 12 338 records identified, 121 were globally eligible. Thirty-three high-income country studies were included in this article (29 quantitative and 4 qualitative, with 33 252 participants). Cross-country variability was found in knowledge, beliefs and behaviours. Misunderstandings were prominent around the definition of AMR and its implications for health. Narrative gender differences were inconsistent and qualitative data were limited, but gave insight into potential drivers of behaviours.

**Conclusions:**

Despite international variations, knowledge gaps, misconceptions and counterproductive behaviours were widespread. Findings show that AMR is a system-wide issue that requires attention from all One Health stakeholders, and a unified, multi-faceted, multidisciplinary approach that can help tackle the continuing public health threat, and change behaviour to protect health.

## Introduction

Antimicrobial resistance (AMR) is a growing public health concern as bacteria evolve and evade human intervention efforts. In 2019 AMR is reported to have directly caused 1.27 million deaths, and contributed to 4.95 million deaths,^1^ with 10 million annual attributable deaths predicted globally by 2050.^2^ Currently, AMR is making everyday infections, e.g. skin infections and post-surgery infections, harder to treat and complicating vital life-saving procedures, such as cancer treatments and surgical procedures, due to the lack of preventive infection and treatment options.^3^ AMR poses a direct threat to the United Nations Sustainable Development Goals, undermining commitments to *good health and well-being*, *poverty reduction* and *reduced inequalities.*^4^ Public knowledge, beliefs and behaviours significantly contribute to, and are argued to be at the forefront of, the continual evolvement of AMR. These include knowledge of methods for addressing AMR, beliefs concerning the scale of the problem, beliefs about personal relevance,^5^ hygiene behaviours,^2^ and the misuse of antibiotics.^6^ Combatting AMR requires a multifaceted approach, involving behaviour change in healthcare workers, patients, caregivers, livestock farmers and those who sell antibiotics.^7,8^ Effective interventions are needed to prevent the spread of infection and disease, drawing on what we know about individual and public behaviours.^9^ Previous empirical research on public knowledge, beliefs and behaviours related to AMR has focused on antibiotic use,^10–12^ are country or continent-specific,^13–16^ or are intervention assessments rather than baseline measures.^17–19^

Discourse around AMR in low- and middle-income countries (LMICs) focuses on concerns such as non-prescription antibiotic use,^20^ poor sanitation, or lack of access to clean water and healthcare.^21–23^ AMR is also a concern in high-income countries (HICs) due to factors including high rates of national antimicrobial usage in primary and secondary care and MDR organisms. A global spatial modelling study found the national median (Mdn) antibiotic usage in HICs was 72% (IQR = 60–78), much higher than that of LMICs (Mdn = 42%, IQR = 35–49).^24^

AMR has been described as a multi-faceted phenomenon that does not recognize geographical borders,^8^ emphasizing the benefits of a global perspective on the ability to inform, create and implement universally applicable strategies to address AMR.^25,26^ Furthermore, gender inequalities have been shown to contribute to susceptibility and experience of AMR infections. In this context, the WHO defines ‘sex’ as ‘the biological characteristics that define humans as male or female’^27^ and gender as ‘the characteristics of women, men, girls and boys that are socially constructed’.^28^ In the context of this review, we use the binary categories of male and female as this is typically how gender is reported in AMR literature,^29^ while recognizing that gender encompasses diverse identities beyond these categories. Reported gender inequalities in AMR include evidence that men are more susceptible to infections than women, whereas women often experience more heightened symptoms once infected,^30^ leading towards critical and long-term impact. The WHO explains that AMR national action plans (NAPs) often overlook the influence of gender.^31^ Gender differences in key cognitions like knowledge, beliefs and subsequent behaviours related to AMR remain underreported. A recent systematic review echoes these gaps, highlighting insufficient research on the role of gender in antibiotic use.^32^ Similarly, a 2021 scoping review of gender differences in knowledge and practices related to antibiotic use in Southeast Asia further underscores the importance of contextual variances when considering how gender shapes antibiotic use.^33^ Three reviews have collectively had a global focus on a broad range of public knowledge and beliefs related to AMR, synthesizing studies up to 2018, but did not explore gender differences.^10,34,35^ Since this time, there have been additional challenges such as the coronavirus disease 2019 (COVID-19) pandemic, which may have accelerated the negative impacts of AMR,^36^ and changed the public health landscape.

This systematic review aims to provide a comprehensive, up-to-date synthesis of qualitative and quantitative research in HICs on public knowledge, behaviours and beliefs regarding AMR. The research questions are: (i) What are the gaps in public knowledge regarding AMR? (ii) What misconceptions do people have in their beliefs about AMR and what is the prevalence of these? (iii) What behaviours could be contributing to, or helpful in combating, AMR? and (iv) To what extent are there gender differences in the outcomes?

## Materials and methods

### Protocol and registration

The review protocol was registered on PROSPERO on 2 April 2025 (ID: 584876). The protocol has been updated since then on 1 July 2026 to reflect the split of HICs and LMICs.

### Eligibility criteria

The search included primary qualitative, quantitative or mixed methods studies that measured public knowledge, beliefs and behaviours related to AMR. Studies were included if they reported data from adults aged 18 years or over from the general population or public only (studies of patients attending primary care, outpatient clinics, or admitted for non-infectious, non-AMR specific reasons were included). Exclusion criteria included non-English language studies; unpublished studies; those within specific population groups (e.g. healthcare professionals, parents or caregivers, those with specific health conditions); intervention studies; studies not involving human participants (e.g. animal studies, lab-based microbiological research); studies without primary data (e.g. editorials, commentaries, reviews, opinion pieces, letters to the editor, conference abstracts without full data, protocols without results); studies using only clinical, epidemiological or microbiological outcome measures (e.g. prevalence of resistant organisms) without any assessment of affect, cognitions or behaviours; studies without general population data reported separately or focusing solely on healthcare professionals, students in health disciplines, or prescribers; and duplicate publications or studies with overlapping data, unless they reported unique or additional findings.

### Search strategy

Initial searches were carried out on 11 databases/registers including Web of Science Core Collection, Medline and PsycINFO for studies published between August 2014 and December 2024. Searches were re-conducted on the 12 June 2026 prior to the final analysis, with strings identical to the original search to ensure any new studies were included in the review. Supplementary file S1 (available as Supplementary data at *JAC-AMR* Online) shows the full search strategy.

### Study selection

All studies’ titles, abstracts and full texts were independently double screened by 11 reviewers (C.S., E.B., L.J.S., A.F., C.C.K., I.L., R.F., T.E., M.D., N.S., G.W.S.). For (i) title and abstract, and (ii) full-text screening, Cohen’s kappa ranged from: (i) −0.02 to 1.00, Mdn = 0.32; and (ii) 0.16 to 0.93, Mdn = 0.77. The mean percentage of agreement was (i) 91.94% (range = 50.00%—99.37%), and (ii) 87.94% (range = 59.26%—96.77%). At each stage, the review team were reminded of the inclusion and exclusion criteria before the next stages, and any authors requiring additional support had a meeting with C.S. to address any queries. Any disagreements at both stages were resolved by a third reviewer.

### Data extraction

A data extraction template form and instructions were created by C.S. and piloted by T.E. and I.L. before use by the research team to ensure usability and consistency. Data were then double extracted (C.S. independently extracted from all included studies and nine other reviewers independently extracted a random proportion for the second extraction (E.B., L.J.S., A.F., I.L., R.F., T.E., N.S., M.D., E.M.). The Mixed Methods Appraisal Tool (MMAT)^37^ was used to appraise the quality of each paper. Data extraction included data relating to: *methods* (i.e. date of data collection, country and continent where data were collected, method of data collection, sampling method, and measures); *sample characteristics* [i.e. total (*N*), gender (*n*, %), age (mean, SD and range) and education level (*n*, %)]; and *outcomes* (i.e. knowledge, beliefs, behaviours and gender differences, where the measure was noted along with corresponding statistical results (*n* or percent prevalence, ORs, adjusted ORs, CIs, *P* values). These were extracted from the Results section of the papers and/or the Supplementary materials. Any differences in extracted data were resolved through discussion.

### Data synthesis

Data were synthesized in a descriptive, non-meta-analytic manner at study- and country-level in Microsoft Excel and IBM^®^ SPSS^®^. This unweighted approach was taken due to the heterogeneity in population characteristics, measures and definitions of outcomes. C.S. and T.E. categorized extracted items into knowledge, beliefs, behaviours and gender differences based on the underlying construct rather than the original authors’ categorizations. Items were categorized as *knowledge* if they had an objectively correct/incorrect answer (verified by reference to literature) or *beliefs* if they were measuring a subjective preference or attitude. Synthesized knowledge results included the percentage of participants who provided incorrect responses, or responses that showed a lack of awareness (e.g. answered ‘no’ to having heard of a certain term). The same synthesis and logic were applied to beliefs to determine misconceptions. For the behaviour outcome, the included data reflect the percentage of participants who reported that they have or would engage in a behaviour, deemed by ‘yes/true/strongly agree/agree/always/often/sometimes’ answers (see Supplementary file S2 for the coded direction of each item). Knowledge, belief and behaviour synthesized items were then grouped into domains with others that measured similar constructs. Behaviour items were labelled as ‘positive’ (acceptable behaviours that would not contribute to AMR) and ‘negative’ (behaviours that could contribute to AMR). A mean of each grouping was computed and analysed using descriptive statistics with the number of studies (*k*), mean, SD, Mdn and range reported, where medians were examined when distributions were highly skewed. Due to the non-meta-analytic approach, the calculated mean and Mdn should be interpreted as a descriptive summary as opposed to a pooled prevalence estimate. Studies contributed to a domain even if they measured only one item. Gender differences could not be meta-analysed as the few studies that reported gender differences had measures that were too heterogeneous. Therefore, we summarized gender differences narratively.

Given the large number of result domains, we applied a reporting threshold whereby only (i) domains or sub-domains that included results from at least three studies and (ii) domain items that were supported by more than one study were included in the narrative synthesis. All domains, regardless of the number of contributing studies, are reported in the summary table. Qualitative data were included only if there were extractable participant quotes. Quotes were then analysed narratively using deductive thematic analysis, with each quote coded under the relevant outcome. PRISMA guidelines^38^ were followed throughout to enable a structured process and allow for transparency.

## Results

At the point of completion of data extraction, a total of 121 global studies were deemed eligible. Due to this high volume and the aforementioned delineation between HICs’ and LMICs’ contextual differences in AMR, antibiotic use and healthcare challenges, and the majority of AMR discourse relating to LMICs, only studies where data collection took place in an HIC country are included in this article (per World Bank classifications for the fiscal year 2026).^39^ Studies conducted in LMICs will be presented in a second article (see Figure 1 for the PRISMA flow diagram summarizing study identification, screening and inclusion).

**Figure 1.**
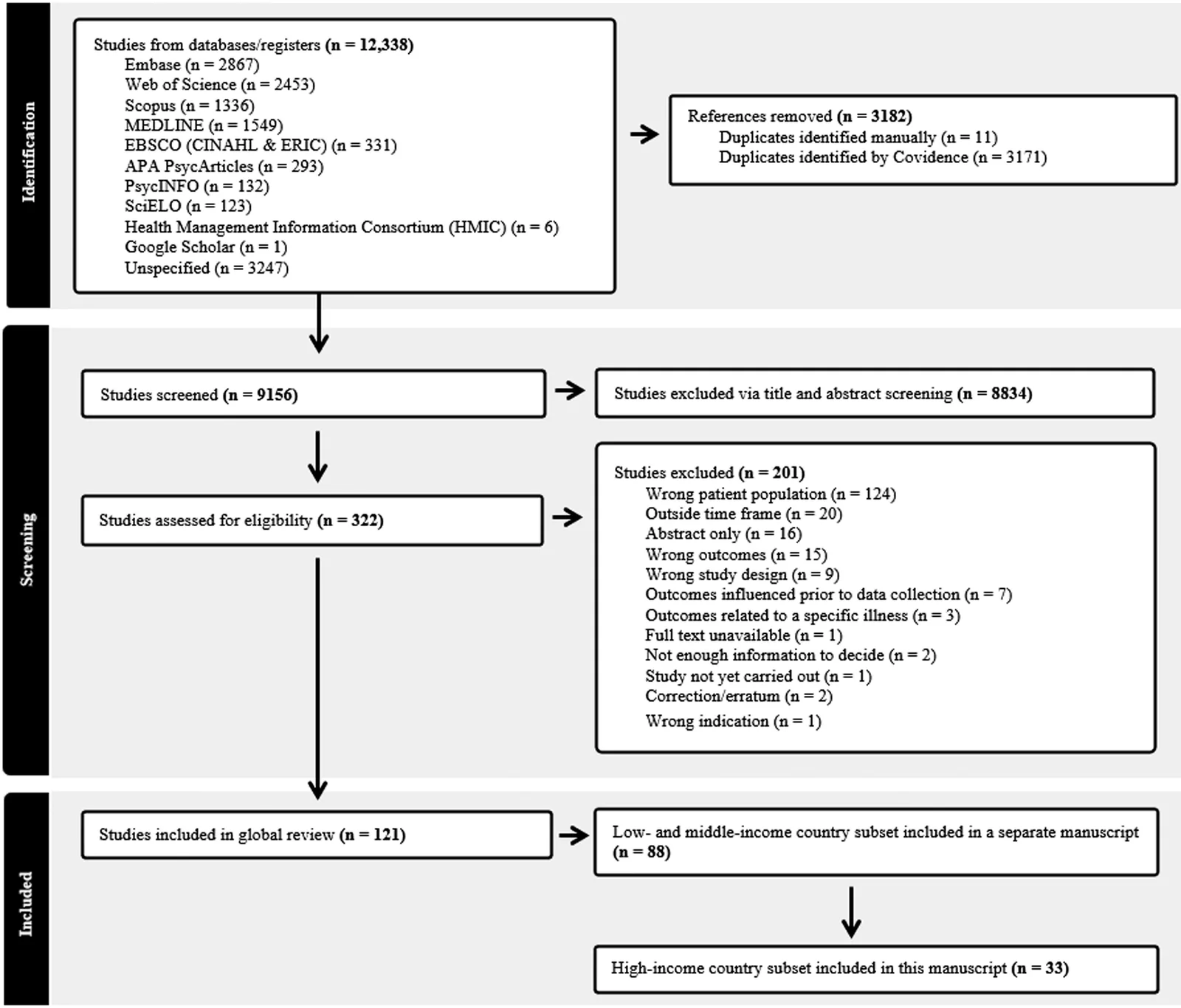
PRISMA flow diagram summarizing study identification, screening and inclusion in a systematic review of public knowledge, beliefs and behaviours related to antibiotics and antimicrobial resistance in high-income countries.

### Study and sample characteristics

There were 33 studies included for review (29 quantitative and 4 qualitative). Three studies reported multiple independent results (i.e. country-specific findings, variable response rate findings, or sub-population results); therefore the number of data points contributing to the summary tables or narrative results may exceed the total number of included studies. We refer to *k* throughout as the number of studies. One study^40^ was conducted by members of the review team; however, standard procedures for data extraction and synthesis were followed, including independent extraction by a reviewer who was not involved in that study, to minimize potential bias. Table 1 provides a summary of included studies’ characteristics.

**Table 1.** Characteristics of studies and samples included in a systematic review of public knowledge, beliefs and behaviours related to antibiotics and antimicrobial resistance in high-income countries (*k* = 33)

Study characteristics							Sample characteristics						
Data collection						Gender, %						Education,^a^ %	
Author (year published)	Year	Country	Method	Sampling	Measures	Outcomes extracted	Sample size	Male	Female	Other	Age range, y	Pre-university level	University level
Adam & Bruce (2023)^41^	2023	UK	Survey	Probability	Based on previous literature alongside working group	(1) Knowledge (2) Beliefs (3) Behaviours (4) Gender differences	5693	N/A^b^	N/A^b^	N/A^b^	18–>55	N/R	N/R
Al Saleh, *et al.* (2021)^42^	2020	Saudi Arabia	Survey	Non-probability	Developed by research team	(1) Knowledge (3) Behaviours	843	29.50	70.50	N/A	18–66	22.30	77.70
Albadrani *et al.* (2023)^43^	2023	Saudi Arabia	Survey	Non-probability	Developed in reference to previous literature and guidelines	(1) Knowledge (3) Behaviours (4) Gender differences	2067	35.02	64.97	N/A	18–>45	24.48	75.52
Alhur *et al.* (2024)^44^	2024	Saudi Arabia	Survey	Non-probability	Based on previous literature	(1) Knowledge (2) Beliefs (3) Gender differences	1561	31.52	68.48	N/A	N/R	16.46	83.54
Alnasser *et al.* (2021)^45^	2020	Saudi Arabia	Survey	Non-probability	Based on previous literature	(1) Knowledge (2) Beliefs (3) Behaviours (4) Gender differences	443	30.20	69.00	N/A	18–>44	29.40	70.70
Alshelwi (2018)^46^	2016	Saudi Arabia	Survey	Non-probability	Based on opinion and advice from relevant professionals	(1) Knowledge (2) Beliefs	654	55.50	44.50	N/A	18–>40	52.12	47.85
Ancillotti *et al.* (2018)^47^	2016	Sweden	Focus groups	Non-probability	Developed by research team	(2) Beliefs	23	56.52	43.48	N/A	20–81	52.00	48.00
Anderson (2022)^48^	2018	UK (England, Scotland, Wales)	Survey	Probability	Wellcome Monitor Wave 4 survey	(2) Beliefs (3) Behaviours (4) Gender differences	2708	48.70	51.30	N/A	18–>70	64.00	36.10
Bdair *et al.* (2024)^49^	2023	Saudi Arabia	Survey	Non-probability	Developed by research team	(1) Knowledge (2) Beliefs (3) Behaviours (4) Gender differences	600	53.50	46.50	N/A	<30– ≥ 41	16.20	83.80
Benmerzouga *et al.* (2019)^50^	2016–2017	Saudi Arabia	Survey	Non-probability	Developed by research team	(1) Knowledge (3) Behaviours	500	43.00	57.00	N/A	N/R	37.00	62.00
Bhat *et al.* (2023)^51^	2023	Saudi Arabia	Survey	Non-probability	Developed by research team	(1) Knowledge (2) Beliefs	300	33.30	66.70	N/A	18–80	27.00	73.00
Boboia *et al.* (2020)^52^	2016	Romania	Survey	Probability	Developed by research team	(1) Knowledge (2) Beliefs (3) Behaviours	301	33.00	67.00	N/A	18–>60	33.00	67.00
David *et al.* (2022)^53^	2020	France	Survey	Non-probability	Developed by research team	(1) Knowledge (3) Behaviours	1104	48.00	52.00	N/A	18–85	N/R	N/R
Davis *et al.* (2023)^54^	2017–2018	Australia	Interview	Non-probability	Developed by research team	(1) Knowledge (2) Beliefs (3) Behaviours	99	41.40	58.58	N/A	18–99	N/R	N/R
El Zowalaty *et al.* (2016)^55^	2014–2015	Saudi Arabia	Survey	Non-probability	WHO Antibiotic Resistance Multi-Country Public Awareness Survey and previous literature	(1) Knowledge (3) Behaviours (4) Gender differences	1149	41.00	59.00	N/A	N/R	47.30	52.70
Guo *et al.* (2022)^56^	2020–2021	Singapore	Survey	Probability	WHO Antibiotic Resistance Multi-Country Public Awareness Survey	(1) Knowledge (2) Beliefs (3) Behaviours (4) Gender differences	2004	48.40	51.60	N/A	21–>65	64.30	35.70
Ilktac *et al.* (2020)^57^	N/R	Cyprus	Survey	Probability	WHO Antibiotic Resistance Multi-Country Public Awareness Survey	(1) Knowledge (2) Beliefs (3) Behaviours	701	51.20	48.80	N/A	18–>65	49.90	50.10
Kamata *et al.* (2018)^58^	2017	Japan	Survey	Probability	European Commission Special Eurobarometer 445: Antimicrobial Resistance questionnaire and from previous literature	(1) Knowledge (2) Beliefs (3) Behaviours (4) Gender differences	3390	51.20	48.80	N/A	20–29	65.00	35.00
Kharaba *et al.* (2024)^59^	2022	United Arab Emirates	Survey	Non-probability	WHO Antibiotic Resistance Multi-Country Public Awareness Survey and previously validated instrument used in previous studies	(1) Knowledge (2) Beliefs (3) Behaviours	1074	24.67	75.33	N/A	18–69	40.59	59.40
Lajunen *et al.* (2023)^60^	N/R	Greece	Survey	Non-probability	Based on previous literature	(1) Knowledge (2) Beliefs (3) Behaviours	309^c^	49.00	49.40	N/A	N/R	16.50	83.10
Lee *et al.* (2023)^61^	2020	Singapore	Survey	Non-probability	Created by research team and taken from previous studies	(1) Knowledge (3) Behaviours (4) Gender differences	967	49.10	50.80	N/A	21–>60	44.50	55.40
Lescure *et al.* (2022)^62^	2019	The Netherlands	Focus groups	Non-probability	Based on previous literature	(1) Knowledge (2) Beliefs (3) Gender differences	64	31.25	68.75	N/A	28–84	82.81	17.19
Lim *et al.* (2021)^63^	2019	Singapore	Survey	Non-probability	Based on previous literature	(1) Knowledge (2) Beliefs (3) Behaviours (4) Gender differences	706	43.30	56.70	N/A	22–86	55.80	43.60
McCracken *et al.* (2023)^64^	2018	USA	Survey	Non-probability	N/R	(1) Knowledge (2) Behaviours	657	N/R	78.00	N/A	N/R	21.00	78.00
Papadimou *et al.* (2022)^65^	2021	Greece	Focus groups	Non-probability	Adapted from previous study	(1) Knowledge (2) Beliefs (3) Behaviours	20	40.00	60.00	N/A	21–55	10.00	90.00
Pennino *et al.* (2023)^66^	2022	Italy	Survey	Non-probability	Developed by health professionals	(1) Knowledge (2) Beliefs (3) Behaviour (4) Gender differences	1158	41.36	58.64	N/A	N/R	69.43	30.57
Raupach-Rosin *et al.* (2019)^67^	2014–2015	Germany	Survey	Probability	Based on previous literature	(1) Knowledge (2) Beliefs (3) Behaviours (4) Gender differences	872	N/R	60.20	N/A	N/R	57.20	42.80
Schmiege *et al.* (2022)^68^	2020	Germany	Survey	Probability	Developed by research team	(1) Knowledge (2) Beliefs (3) Behaviours	158	49.93	49.47	0.60	N/R	36.71	61.39
Smith & Buchan (2023)^69^	2020–2021	UK	Survey	Non-probability	Developed by research team	(1) Knowledge (2) Beliefs (3) Behaviours (4) Gender differences	164	38.40	61.60	N/A	18–>65	50.00	50.00
Vallin *et al.* (2016)^70^	2013	Sweden	Survey	Non-probability	Based on previous literature	(1) Knowledge (2) Beliefs (3) Gender differences	1426	43.60	56.40	N/A	18–74	53.70	46.30
Worthington *et al.* (2020)^71^	N/R	USA	Survey	Non-probability	N/R	(2) Beliefs	1014	N/R	71.30	N/A	21–84	28.40	49.50
Osman *et al.* (2025)^72^	2023–2024	Oman	Survey	Non-probability	Developed based on validated instruments from previous studies and expert consultation	(1) Knowledge (2) Beliefs (3) Behaviours	155	41.30	58.7	N/A	N/R	37.4	62.6
Shields *et al.* (2026)^40^	2024	Republic of Ireland and Northern Ireland	Survey	Non-probability	WHO Antibiotic Resistance Multi-Country Public Awareness Survey and questions on ESKAPE pathogens derived from literature	(1) Knowledge (3) Behaviours	Republic of Ireland: 396 Northern Ireland: 415	Republic of Ireland: 49 Northern Ireland: 49.30	Republic of Ireland: 50.50 Northern Ireland: 50	Republic of Ireland: 0.50 Northern Ireland: 0.70	18–>65	Republic of Ireland: 54.20 Northern Ireland: 50.60	Republic of Ireland: 45.70 Northern Ireland: 49.40

*k*, number of studies; N/R, not reported, whereby it was expected to see these data reported but they were missing from the manuscript; N/A,  not applicable, whereby the data were not expected to be in the manuscript.

^a^Education refers to the highest level of education attained at the point of data collection, which was organized into pre-university and university level.

^b^Gender breakdown reported was not representative of the whole sample and was therefore excluded from our analysis.

^c^Study also collected data in Turkey; these were excluded due to Turkey not being classified as a high-income country.

Most studies used non-probability sampling techniques (*k* = 25, 75.76%), surveys for data collection (*k* = 29, 87.88%), and were carried out in Asia (*k* = 16, 48.48%) or Europe (*k* = 14, 42.42%). There were slightly more studies whose data collection took place between 2020 and 2024 (*k* = 16, 48.48%) compared with 2013–2019 (*k* = 14, 42.42%). Nearly one-third of studies were carried out in Saudi Arabia (*k* = 9, 27.27%). Other countries included the UK (*k* = 3, 9.09%) and the USA (*k* = 2, 6.06%). Most studies used only measures developed by the research team/by reference to previous literature (*k* = 22, 66.67%) rather than reliable and validated scales. There were 33 252 participants across all studies, with the proportion of female participants ranging from 43.48% to 78.00% (Mdn: 58.58, IQR: 50.40–66.85, *k* = 32), and male participants ranging from 24.67% to 56.52% (Mdn: 43.15, IQR: 34.59–49.15, *k* = 29). The proportion of participants who were educated up to pre-university level ranged from 10% to 82.81% (Mdn: 42.55, IQR: 27.00–54.20, *k* = 30) and up to university level it was 17.19% to 90% (Mdn: 52.70, IQR: 45.70–73.00, *k* = 30).

### Outcome 1: Knowledge

#### Knowledge of AMR

Knowledge (mean, SD) was lowest around the existence of AMR (see Table 2). Of those studies, 83.13% (SD = 10.65) incorrectly thought people can become resistant to antibiotics (range = 62.00%–92.00%, *k* = 9), and 22.10% (SD = 28.57) did not know bacteria can become resistant to antibiotics (range = 6.00%–77.37%, *k* = 5). Lack of awareness of terms was seen mostly with ‘AMR’ (mean = 80.53, SD = 5.78, range = 76.00–89.00, *k* = 4), followed by ‘antimicrobial resistance’ (mean = 58.50, SD = 0.42, range = 58.00–59.00, *k* = 4) and ‘antibiotic resistance’ (mean = 33.48, SD = 13.09, range = 15.70–53.40, *k* = 7). With regards to causes of AMR, 47.93% (SD = 22.58) did not perceive the overuse or use of antibiotics in livestock as a cause (range = 4.30–69.50, *k* = 6), and 28.52% (SD = 16.45) did not attribute the inappropriate use of antibiotics in humans as a cause of AMR (range = 3.66–67.50, *k* = 12). Regarding the consequences of AMR, 31.40% (SD = 22.57) were not aware of its threat to medical procedures (range = 5.00–55.30, *k* = 6), and 34.72% (SD = 11.53) were not aware of its threat to health (range = 25.00–54.70, *k* = 5). Country-level descriptive analysis showed that a study carried out in Japan had the highest level of incorrect knowledge of AMR, with an average of 70.85% answering questions incorrectly (*k* = 1), followed by those carried out in Cyprus (mean = 62%, *k* = 1) and Singapore (mean = 55.18, SD = 7.97, Mdn = 51.98, range = 49.31–64.25, *k* = 3). Studies conducted in Saudi Arabia contributed the most evidence to this domain, with an average of 39.01% (SD = 14.50) answering questions incorrectly (Mdn = 30.39, range = 28.70–62.85, *k* = 6) (see Supplementary file S3 for a full breakdown of country-level sub-domain results).

**Table 2.** Descriptive statistics results for participants in high-income countries’ average number of people who provided answers that displayed incorrect knowledge, belief misconceptions, and engagement in behaviours related to antibiotics and antimicrobial resistance (*k* = 33)

Outcome	Domains and sub-domains	*k*	Mean^a^	SD^b^	Mdn	Min.	Max.
**Knowledge**	**Domain 1: Knowledge of AMR**	21	43.96	14.44	46.93	13.3	70.85
	Sub-domain 1. Causes of AMR	13	39.35	20.72	44.18	3.98	80.7
	Sub-domain 2. Heard of terms	9	44.09	17.11	44.69	5.1	64.25
	Sub-domain 3. Consequences of AMR	11	36.9	13.42	31.77	20.8	55.3
	Sub-domain 4. Existence of AMR	13	54.64	18.79	58.29	20.92	90
	Sub-domain 5. Tackling AMR	4	35.62	21.78	43.2	4	52.08
	**Domain 2: Knowledge of antibiotics**	23	36.36	18.17	33.65	9.87	93
	Sub-domain 1. Appropriate antibiotic use	15	24.24	14.18	18.5	9.6	61.03
	Sub-domain 2. Antibiotic purposes and effects	19	45.93	16.76	44.17	25.57	93
	Sub-domain 3. Appropriate antibiotic prescription	1	44.47	—	—	—	—
	Sub-domain 4. Identification of antibiotics	1	37.02	—	—	—	—
**Beliefs**	**Domain 1: AMR risk perception**	7	40	15.75	38.9	19.66	64.1
	**Domain 2: Attribution of responsibility**	4	21.9	11.06	20.77	10.3	35.77
	**Domain 3: Antibiotic use**	2	44.45	11.53	44.45	36.3	52.6
	**Domain 4: Expectations and preferences**	6	27.19	16.42	23.95	8.67	57.3
	**Domain 5: Trust**	3	27.49	35.96	7.45	6.02	69
	**Domain 6: Self-efficacy**	3	19.03	13.09	13.4	9.7	34
	**Domain 7: Response efficacy**	1	72.2	—	—	—	—
**Behaviours**	**Domain 1: Antibiotic obtainment**	18	—	—	—	—	—
	Sub-domain 1. Positive behaviours	9	70.22	28.89	75.6	23	99.48
	Sub-domain 2. Negative behaviours	15	20.43	19.17	11	0.95	63.6
	**Domain 2: Antibiotic use**	25	—	—	—	—	—
	Sub-domain 1. Positive behaviours	17	68.23	24.59	79.63	26.44	95.7
	Sub-domain 2. Negative behaviours	22	28.73	20.69	21	0.3	64
	**Domain 3: Antibiotic disposal**	4	—	—	—	—	—
	Sub-domain 1. Positive behaviours	4	22.7	28.21	13.06	0.7	64
	Sub-domain 2. Negative behaviours	3	29.27	9.1	25.1	23	39.71
	**Domain 4: Information seeking**	4	—	—	—	—	—
	Sub-domain 1. Positive behaviours	4	47.99	33.37	38.81	19.1	95.26
	Sub-domain 2. Negative behaviours	2	28.4	1.98	28.4	27	29.8

*k*, number of studies; Mdn, median percentage of study respondents; Min., minimum number of study respondents; Max., maximum number of study respondents.

^a^Mean is of the percentage of study respondents who provided answers that displayed incorrect knowledge, belief misconceptions and engagement in behaviours.

^b^SD of mean percentage of study respondents.

Empty cells (—) represent where one study contributed to the sub-domain yielding no results beyond a mean value. Maximum values in Table 2 reflect study-level averages across belief items; single-item values may be higher.

#### Knowledge of antibiotics

With regards to antibiotic purposes and effects, nearly half answered questions incorrectly. In these studies, 51.98% (SD = 13.48) incorrectly identified that antibiotics were effective against viruses (range = 26.50–78.10, *k* = 12), including colds/flu (mean = 44.05, SD = 23.78, range = 13.40–86.20, *k* = 13), and 39.78% (SD = 23.53) thought they were not effective against bacteria (range = 10.63–93.00, *k* = 11). A moderate knowledge gap was highlighted with 36.11% (SD = 19.85) of people unaware that antibiotics can have side effects (range = 11.00–61.20, *k* = 6) and 30.96% (SD = 11.77) lacking knowledge that antibiotics can kill naturally occurring bacteria (range = 21.00–50.20, *k* = 5). Modest knowledge gaps were found with appropriate antibiotic use, with 23.64% (SD = 22.00) thinking it was acceptable to stop taking antibiotics before finishing the course (range = 9.60–89.80, *k* = 13), and 20.35% (SD = 11.43) believing it was acceptable to share antibiotics (range = 9.33–41.28, *k* = 9). Also, 41.91% (SD = 7.74) thought it was acceptable to buy or request the same antibiotics based on previous use (range = 32.00–48.14, *k* = 4) (see Table 2 for full details).

Country-level descriptive analysis showed that a study carried out in Romania had the highest level of incorrect knowledge of antibiotics, with an average of 93% answering questions incorrectly (*k* = 1), followed by those carried out in Japan (mean = 52.03%, *k* = 1) and Italy (mean = 40.46%, *k* = 1). Studies conducted in Saudi Arabia contributed the most evidence to this domain, with an average of 38.43% (SD = 17.56) answering questions incorrectly (Mdn = 39.57, range = 9.87–58.56, *k* = 8), followed by those carried out in Singapore (mean = 32.64, SD = 13.04, Mdn = 35.27, range = 18.50–44.17, *k* = 3). One study^62^ had relevant qualitative data (see Table 3). A participant described their understanding of the dangers of stopping an antibiotic too early informed by a correct understanding of AMR: ‘*When I stop the antibiotic treatment too quickly, bacteria in my body will become stronger and the next time these bacteria will not be defeated by the same antibiotics*.’ Another participant spoke of their correct understanding of AMR: ‘*It is not only for yourself, because bacteria in general are becoming resistant against antibiotics, so [*  *. . .*  *] there is an increase in super bacteria for which we only have limited effective medicines.*’

**Table 3.** Qualitative AMR data themed under knowledge, beliefs and behaviours outcomes (*k* = 4)

Outcome	Themes	*n*	*k*
Knowledge	Inappropriate use of antibiotics	2	1
	Understanding of AMR as a concept	2	1
	Knowledge of antibiotic side effects	1	1
Beliefs	Perceived severity	4	2
	Perceived susceptibility	3	2
	Perceived benefits	10	3
	Perceived barriers	17	3
	Self-efficacy	7	3
	Cues to action	7	2
Behaviours	Demanding or requesting antibiotics	6	1
	Availability of antibiotics	1	1
	Finishing an antibiotic course early	2	1
	Avoiding using antibiotics	1	1
	Experience of side effects	1	1
	Information seeking	4	1
	Using antibiotics without a prescription	1	1
	Alternatives to medicine	2	1
	Using leftover antibiotics	1	1

*k*, number of studies that had quotes relevant to each theme; *n*, number of quotes that fit under the theme.

### Outcome 2: Beliefs

Misconceptions were high in relation to AMR risk perception; 56.69% (SD = 26.12) thought antibiotic resistance is only an issue for people who take antibiotics regularly (range = 27.66–78.30, *k* = 3) and 41.96% (SD = 31.53) thought it did not impact their own and their families’ health (range = 19.66–64.25, *k* = 2). With regards to trust, 37.51% (SD = 44.53) did not trust that healthcare professionals were prescribing antibiotics appropriately (range = 6.02–69.00, *k* = 2). With expectations and preferences, 16.70% (SD = 9.48) expected an antibiotic when they go to the doctor with a cold or flu (range = 10.00–23.40, *k* = 2). Misconceptions were least common with regards attribution of responsibility: only 14.17% (SD = 5.14) believed that individuals are not responsible for taking antibiotics correctly (range = 10.30–20.00, *k* = 3) (see Table 2). A study conducted in Cyprus reported the highest level of misconceptions, with an average of 68.15%. Studies conducted in Saudi Arabia contributed the most evidence to the beliefs outcome, with moderately low levels of misconceptions (mean = 34.56, SD = 16.55, Mdn = 34.00, range = 17.21–57.30, *k* = 5), followed by those conducted in the UK (mean = 30.26, SD = 23.85, Mdn = 30.26, range = 13.40–47.13, *k* = 2), Germany (mean = 29.71, SD = 10.78, Mdn = 29.71, range = 22.08–37.33, *k* = 2) and Singapore (mean = 21.20, SD = 3.11, Mdn = 21.20, range = 19.00–23.40, *k* = 2). One study in Oman reported the lowest level of misconceptions, with an average of 9.70% prevalence.

Three studies collected relevant qualitative data^47,62,65^ (see Table 3). Discussions focused largely on barriers to using antibiotics appropriately. At an individual level, participants in the 2022 study by Lescure *et al.*^62^ described feeling pressure to exaggerate symptoms to obtain antibiotics: ‘*You feel angry and desperate, and you do not know what to do. Then, once in a time, you will exaggerate a bit to receive the antibiotics you want.*’ Difficulty in obtainment was echoed in the same study where participants described GPs’ reluctance to prescribe antibiotics: ‘*I was furious because I did not receive antibiotics easily. You only receive antibiotics after a week, after severe worsening of [your child’s] symptoms. It isn’t easy.*’ On the other hand, in the 2018 study by Ancillotti *et al.*,^47^ barriers were discussed at a system level where antibiotics are too easy to obtain: ‘*…too easy to take, from the perspective of being too easy for both a doctor who is a bit fed up with his job and the patient who wants to recover quickly.*’ This was echoed in the 2022 study by Papadimou *et al.*:^65^ ‘*I also think the problem is the rather easy access, because of the cost and because no serious prescription is required*.’ Perceived benefits focused largely on the appropriate use and prescription of antibiotics. A participant in the Lescure *et al.* study^62^ described no longer having a perceived benefit of using antibiotics after a GP advised rest instead of a prescription: ‘*I went to the GP. She did not give me antibiotics and told me to rest and lie down and that the disease would disappear. Yes, then I was angry. But now I understand antibiotics are not good, I never use them.*’ A participant in the study by Papadimou *et al.*^62^ also showed a reluctance to take antibiotics, believing they offer no benefit unless the infection is severe: ‘*[When it is not a life-threatening infection], I would avoid using antibiotics for myself and the people around me*.’ On the other hand, in Lescure *et al.*’s study,^62^ a participant described their desire for antibiotics, believing they would help their child feel better: ‘*I know antibiotic use is better for my children. Here, children are getting sicker*.’ With regards self-efficacy, participants discussed how combating AMR requires effort beyond individual responsibility. One example in the study by Papadimou *et al.*^62^ highlights a participant’s disbelief in being able to make meaningful personal impact: ‘*I only see it as collective. My individual practice is not enough*’.

### Outcome 3: Behaviours

Negative antibiotic obtainment behaviours (mean, SD) included having asked for or demanded antibiotics from the GP, which was seen in 25.18% of people (SD = 22.15) (range = 6.30–59.20, *k* = 8), and having used someone else’s antibiotics in 13.44% (SD = 11.64) (range = 1.20–33.70, *k* = 7). Negative antibiotic use was highlighted with 52.56% (SD = 35.12) who had taken antibiotics when they had a cold or the flu (range = 12.00–72.97, *k* = 3). Few people reported having shared antibiotics with others (mean = 10.21, SD = 9.15, range = 0.40–24.30, *k* = 6), and 24.65% (SD = 19.61) had saved antibiotics to use at home (range = 0.30–60.70, *k* = 12). Negative disposal behaviours were shown with 48.50% (SD = 25.75) who said they would discard leftover antibiotics in domestic waste (range = 23.00–74.49, *k* = 3) (see Table 2). A study conducted in Italy reported the highest level of engagement in negative behaviours (mean = 59.28%), followed by one conducted in Oman (mean = 49.40%). Studies conducted in Saudi Arabia contributed the most evidence to this element, with moderate levels of engagement (mean = 37.53, SD = 16.54, Mdn = 36.02, range = 11.08–62.08, *k* = 10). Three studies in Singapore reported low levels of engagement (mean = 9.87, SD = 7.67, Mdn = 11.40, range = 1.55–16.67, *k* = 3). The lowest levels of engagement were reported by one study carried out in the Republic of Ireland (mean = 1.33%) and one in Northern Ireland (mean = 0.83%). Positive obtainment behaviours were shown with 84.67% (SD = 12.94) taking antibiotics only after medical consultation (range = 75.60–99.48, *k* = 3) and 61.62% (SD = 31.68) obtaining antibiotics via a prescription (range = 23.00–93.60, *k* = 7), but 27.53% (SD = 19.80) said they had taken antibiotics without a prescription (range = 1.30–48.50, *k* = 4).

Positive antibiotic use behaviours showed most people reported taking antibiotics as recommended by a healthcare professional or their instructions (mean = 86.52, SD = 12.90, range = 60.80–95.70, *k* = 6). Also, 57.04% (SD = 21.00) said they would finish the course (range = 35.00–91.00, *k* = 7), but 36.19% (SD = 18.88) reported stopping the course early or as soon as they felt better (range = 10.30–71.10, *k* = 13). Positive disposal behaviours were shown with 25.58% (SD = 27.82) returning leftover antibiotics to a pharmacy (range = 0.70–64.00, *k* = 4). Positive information-seeking behaviours (mean, SD) were shown with 37.62% (SD = 22.11) doing so from healthcare professionals (range = 19.10–62.10, *k* = 3). A study conducted in Northern Ireland reported the highest level of engagement in positive behaviours (mean = 93.60%), followed by one conducted in Greece (mean = 93.50%). Studies conducted in Saudi Arabia contributed the most evidence to this element, with moderate levels of engagement (mean = 63.66, SD = 20.98, Mdn = 70.29, range = 36.57–94.40, *k* = 8). The lowest level of engagement in positive behaviours was reported by one study carried out in Japan (mean = 19.10%).

Two studies reported relevant qualitative data,^62,65^ focusing largely on inappropriate antibiotic use and information seeking (see Table 3). Demanding antibiotics for reasons including relating the symptoms to a previous illness where antibiotics were prescribed is described in the study by Lescure *et al.*:^62^ ‘*When I call the GP and I explain him I have a urinary tract infection, he says “Wait, I first want to see a urine sample.” I say “I have it for the millionth time, so I know how it feels.” I believe it was nonsense to submit a urine sample again in such a situation.*’ One participant in the study by Papadimou *et al.*^65^ described that people casually use antibiotics without professional oversight, when they are deemed beneficial: ‘*Everyone takes antibiotics like caramels and turns to antibiotics without necessarily visiting a physician to prescribe them*.’ Another participant in the Lescure *et al.* study described not using antibiotics and resting instead if ill: ‘*No, I almost never use antibiotics. The same goes for my children. If I am ill, I take a rest and eat a healthy diet. Then, if the illness is not severe, the symptoms will disappear*.’ With regards to information-seeking, a participant in the Lescure *et al.* study explained doing so online but with caution due to potential misinformation: ‘*Then I look on YouTube, but not everything on YouTube is good. I will try things when I believe it is good. But not always the information is good*.’

### Gender differences

Heterogeneous gender outcomes were reported in 15 studies. With regards to knowledge, examples include Alnasser *et al.*,^45^ who reported ‘good’ antibiotic use and AMR knowledge in 91.26% of females and 80.6% of males (χ^2^ = 10.094, *P* = 0.001). Kamata *et al.*^58^ reported mixed knowledge findings: 25.2% of men, compared with 18.3% of women, correctly answered that antibiotics cannot kill viruses. However, 65.4% of women, compared with 38.1% of men, correctly answered that taking antibiotics often has side effects. With regards to beliefs, examples include Smith and Buchan,^69^ who found that males were more likely to choose higher estimates of worldwide AMR-attributable deaths than females (χ^2^, *P* < 0.05). Adam and Bruce^41^ found no significant gender differences in consumer attitudes towards antibiotic use in livestock. With regards to behaviours, examples include Raupach-Rosin *et al.*,^67^ who found that males answered more frequently than females to stopping taking antibiotics as soon as they feel better (15.1% and 5.6% respectively, *P* < 0.001). In addition, El Zowalaty *et al.*^55^ found that female respondents were less likely to self-medicate with antibiotics than males (OR = 0.635, 95% CI = 0.49–0.82).

### Quality appraisal

Of the 29 included quantitative studies, 21 (72.41%) used non-probability sampling. Of these, 10 (47.62%) were rated ‘No’ or ‘Can’t tell’ for the MMAT criterion assessing sampling strategies and representativeness of the population, as opposed to 3 of those that used probability sampling (37.50%). Most of the quantitative studies met the criteria as using appropriate measurements (*n* = 24, 82.76%) and as having statistical analysis appropriate to answering the research question (*n* = 26, 89.66%). Of the four included qualitative studies, all met each MMAT criterion, including appropriateness of qualitative approach and findings being adequately derived from the data. Overall, just over half of studies met five (*n* = 13, 39.40%) or four (*n* = 5, 15.15%) of the MMAT criteria. Of those studies that met four or five criteria, similar numbers used non-probability sampling (*n* = 14, 56%) or probability sampling (*n* = 4, 50%). Across the quantitative studies, the appropriateness of the statistical analysis most frequently met the criterion (*n* = 26, 89.66%), whereas fewer met the criterion for low risk of non-bias (*n* = 13, 44.83%) (see Supplementary file S4 for all MMAT ratings per study per criterion).

## Discussion

This review highlights key knowledge gaps, misconceptions and counterproductive behaviours related to AMR, and inconsistent reporting of and findings related to gender differences, by synthesizing studies published between 2014 and 2026, conducted in HICs (the UK, Germany, Italy, Sweden, USA, Saudi Arabia, Greece, The Netherlands, Australia, Singapore, United Arab Emirates, Cyprus, Japan, France, Romania, Oman, Ireland and Northern Ireland). The findings can inform future research and effective interventions to prevent the spread of infection and disease, drawing on what we know about public behaviours and their determinants.^9^

Misunderstandings and misconceptions were found around the definition, consequences and personal impacts of AMR. We know from conceptual change theory^73^ that misunderstanding basic definitions could prevent understanding of AMR as people build new information based on an incorrect foundation. Therefore, current public health interventions may not be effectively communicating basic AMR-related information, and widespread misunderstandings could be due to the complexity of AMR as a concept. We know from theories of behaviour change, e.g. the Health Belief Model (HBM),^74,75^ that the perceived severity of a health problem, including the societal and personal impacts, can influence behaviour.^76^ This suggests that knowledge, beliefs and behaviours related to AMR are interconnected, and the misunderstandings found could drive optimistic bias and negative behaviours. As a result, interventions aiming to improve knowledge and address misconceptions should prioritize a foundational understanding of AMR and its personal and more general impacts to address these misconceptions. To do so, policy makers and healthcare professionals could use elements that are reported to be successful in interventions. These include the expansion of medical abbreviations,^77^ including ‘AMR’, and the use of multiple communication channels (e.g. television advertising as well as healthcare professional–patient interactions) rather than a single channel.^17^ Long-term evaluations of such interventions are scarce,^17,18^ but the limited evidence showed continued success in some areas, including the reduction of antibiotic prescribing rates.^17^ Beneficial methods of increasing personal relevance to avoid optimistic bias could include written descriptions of members of the public following health recommendations and video footage of people not following recommendations. Interventions that are developed should be evaluated longitudinally to ensure persistent effectiveness and whether the knowledge and beliefs are being retained over time.

In HICs, intervention efforts are shaped by factors such as financial capability to support sustainable campaigns and sufficiently resourced public health infrastructures. Successful AMR interventions in HICs often include mandatory policy enforcement actions and multidisciplinary implementation actors, enabling coordinated messaging across One Health sectors.^78^ This places HICs in an advantageous position to operationalize AMR NAP objectives that aim to improve awareness and understanding of AMR through effective communication, education and training. For example, the NAPs of HICs included in this review such as Saudi Arabia,^79^ Germany^80^ and Sweden,^81^ outline these objectives, and the countries’ funding capacity and implementation resources enable delivery of these interventions at scale, in contrast to LMICs.^78^

Country-specific findings highlighted cross-country and inter-country variability, particularly with regard to incorrect knowledge and misconceptions. Therefore, context-specific factors may be influencing the inherent understanding of AMR, and efforts to reduce misunderstandings and optimistic bias may be inadequate alone. This variability reinforces the interdependence of knowledge and beliefs across settings, and the importance of the WHO stance on tailoring interventions to be context-specific, targeted and adapted to local needs.^82^ Moderating factors of AMR-related cognitions and behaviours are multi-faceted and are influenced by country-specific circumstances such as healthcare systems, culture and social norms.^83^ In HICs in particular, such circumstances could include high healthcare accessibility and cultural expectations around rapid symptom relief. Researchers, policymakers and governing bodies should encourage and fund country-specific research to enable the development of tailored NAPs to incite meaningful AMR-related behaviour change.^82^

There was a lack of awareness of the implications of the overuse or use of antibiotics in livestock as a cause of AMR, and disposing of antibiotics in the domestic waste, which can be a pollutant. These findings highlight a lack of awareness of the agricultural and environmental elements of AMR, and their reciprocal relationship with human health. This emphasizes the need for a One Health approach to addressing AMR,^73^ which is unified, multi-faceted, and multidisciplinary, aiming to improve understanding of AMR and encourage integrated behaviour change across health, agriculture, veterinary and environmental sectors.^2,7,82,84,85^ This is supported by a 2025 systematic review, which found that 92% of successful antimicrobial stewardship (AMS) programmes featured multi-disciplinary elements.^86^

The MMAT highlighted systematic variation in study quality. Studies that employed non-probability sampling techniques showed weaker representativeness and non-response bias compared with those that used probability sampling techniques. Most studies met the criteria for appropriateness of the statistical analysis and measures, and just over half met four or five of all criteria. These findings suggest the internal validity of the results is strong; however, the generalizability of the descriptive estimates should be interpreted with caution due to sampling techniques. While the MMAT highlighted that most studies’ measurements were appropriate at study-level (*n* = 28, 84.85%), there was no standardized or reliable set of dependent variables measured across studies, resulting in difficulty of comparison at review level. Due to this and the problems with variance in outcomes, we were unable to accurately analyse gender differences despite intending to do so, and limited qualitative findings were inconsistent. A core outcome set may be useful as with other fields.^87,88^ In addition, variance was found in the gender distribution of participants, which may influence the generalizability of findings. NAPs could benefit from applying a gender lens to understand and address context-specific influences on AMR knowledge, beliefs and behaviours.^31^ Also, only two studies included participants who reported an identity other than male or female. This also suggests a lack of consistent appropriate measurement tools in empirical research to include gender-diverse subgroups in the context of AMR. This raises a bigger issue of lack of understanding of what is happening in these populations, and can result in a failure to act for the benefit of trans and gender-diverse subpopulations.^89,90^ Future research could benefit from more balanced recruitment strategies as a result. Recent research that used an intersectional framework highlights how contextual factors, e.g. access to healthcare and cultural norms, adjust gender patterns in antibiotic use.^29^ Therefore, it could also be beneficial for future research to consider intersectional approaches to gather a more holistic view of how gender and other personal characteristics influence public knowledge and behaviours related to AMR.

Pooled percentages were unweighted across studies and data were analysed descriptively, meaning all studies contributed to the synthesis equally, regardless of sample size and study quality. This has limitations in that the findings should be interpreted with the consideration that figures may not accurately reflect the overall prevalence of knowledge gaps, misconceptions and behaviours. This approach was appropriate given the heterogeneity of study characteristics. For example, outcome definitions including knowledge and beliefs are often defined in similar ways; statements on the severity of AMR could be argued to have a justifiable, objective, correct answer, but can also be framed as a belief. Items were also measured differently across studies, with variability in responses such as ‘yes/no/do not know’ and ‘strongly agree to strongly disagree’. Therefore, the findings should be interpreted with consideration that measures were organized at the authors’ interpretation. The range of percentages were added to aid the assessment of heterogeneity and extent of knowledge gaps, misconceptions and engagement in behaviours. Despite this, results provide an estimate of the prevalence, which is useful to gauge the extent of drivers within the public domain. There was a low Cohen’s kappa value at title and abstract screening stage (Mdn = 0.32). The inclusion criteria were broad, including multiple outcomes with conceptual nuance, which likely attributed to this finding and could have influenced the selection of included and excluded studies. Although agreement improved at the full-text stage, the potential for selection bias should be considered upon interpretation of the findings. We also extracted data on age and education, which were presented to highlight the demographic profile of the participants in the included studies. These data were not synthesized in relation to the outcomes, which is a limitation as potential associations remain unexplored. Associations between these variables and the outcomes were assessed in some of the included studies, such as Ilktac *et al.*,^57^ Guo *et al.*^56^ and Raupach-Rosin *et al.*^67^ We aim to address this limitation in a follow-up review that includes LMICs, to ensure potential subgroup patterns are explored, which could be useful for tailored public health interventions to address AMR.

Qualitative data provided limited context to quantitative findings, including how beliefs about antibiotic obtainment and benefit influence behaviour. Generalization of qualitative findings to all HICs should be made with caution. Future research could aim to focus on qualitative methods; these have been highlighted as helpful in exploring contextual factors that could explain the drivers of intercountry differences and further inform the development of behavioural interventions.^91^

Three similar previous systematic reviews on public knowledge and behaviours around antibiotic use and/or antimicrobial resistance analysed studies up to 2018 and included HICs.^10,34,35^ Collectively, these studies found persistent misunderstandings about the use of antibiotics for colds and flu, the link between misuse of antibiotics and AMR, and the definition of AMR. The alignment of our findings with those of previous reviews highlights that important knowledge gaps related to AMR are widespread on a global scale, suggests a lack of improvement, and reinforces that the public has an inherent misunderstanding of AMR and its implications. This updated review broadens the literature base by integrating mixed methods, qualitative and quantitative studies. We also reported positive AMR-related behaviours that were not captured in the previous reviews, illustrating a more balanced and comprehensive understanding of AMR-related behaviours. This allows the reader to capitalize on strengths in a country whilst considering areas for improvement.

A key strength of this review is that it offers an up-to-date synthesis of public cognitions and behaviours related to AMR from which to develop interventions incorporating a One Health approach. However, this review analyses only HICs, which do not experience the same AMR impacts as those in LMICs.^21–23^ Data on these countries have been extracted and will be synthesized in a separate article. An HIC perspective on AMR remains important due to the high rates of national antimicrobial use^92^ and the misconceptions and counterproductive behaviours highlighted in this review, which also require attention. Another limitation of this review is the exclusion of non-English language studies. This could lead to the potential bias of findings towards English-speaking countries or English-publishing contexts and limit the broader cultural and geographical representativeness of the findings. Future reviews could consider language translation support to reduce this bias.

## Conclusions

Our findings suggest that knowledge gaps, misconceptions and counterproductive behaviours related to AMR in HICs are widespread and vary across countries. This review stresses that AMR is a system-wide issue that requires attention from all One Health stakeholders and a unified, multi-faceted, multidisciplinary approach that can help tackle the continuing public health threat. Public behaviour interventions should consider context-specific factors to effectively address public misconceptions and reduce optimistic bias. Future research should focus on the formation and adoption of standardized, validated and gender-inclusive instruments to assess AMR and antibiotic-related knowledge, and on qualitative methods that can provide context to concerning quantitative findings and inform targeted interventions.

## Supplementary Material

dlag213_Supplementary_Data

## Data Availability

Data and materials are available on the Open Science Framework (https://osf.io/x7mjk). The review protocol was registered on PROSPERO on 2 April 2025 (ID: 584876). The protocol was updated on 1 July 2026 to reflect the split of high-income and low- and middle-income countries. Details are available in the additional information section of the published protocol.
