# Supplementary material for: Public knowledge, beliefs and behaviours related to antimicrobial resistance and antibiotics: a systematic review of studies in high-income countries
Source: JAC Antimicrob Resist. 2026 Sep 30;8(5):dlag213. doi: 10.1093/jacamr/dlag213 (PMC13624658; doi:10.1093/jacamr/dlag213)
Supplement: Supplementary file 1 [file dlag213_supplementary_data.zip › Supplementary file 1 - Search strategy.docx]

**Search strategy**

1	exp Health Knowledge/

2	(know* or understand*).mp. [mp=title, abstract, heading word, table of contents, key concepts, original title, tests & measures, mesh word]

3	1 or 2

4	exp Awareness/

5	(aware* or percept* or perceiv*).mp. [mp=title, abstract, heading word, table of contents, key concepts, original title, tests & measures, mesh word]

6	4 or 5

7	exp Attitudes/

8	(attitude* or belie* or perception* or “risk perception*” or “self efficacy*” or motivat* or “social norm*” or intention* or “risk taking”).mp. [mp=title, abstract, heading word, table of contents, key concepts, original title, tests & measures, mesh word]

9	7 or 8

10	exp Behavior/

11	(behavio* or practic*).mp. [mp=title, abstract, heading word, table of contents, key concepts, original title, tests & measures, mesh word]

12	10 or 11

13	exp Drug Resistance/

14	(antimicrob* or antibiotic or AMR or antimicrobial resistan* or superbug* or antibacter* or microb* or anti-bacterial agent* or bacterial).mp. [mp=title, abstract, heading word, table of contents, key concepts, original title, tests & measures, mesh word]

15	13 or 14

16	(public* or population* or people* or communit* or societ* or consumer* or patient*).mp. [mp=title, abstract, heading word, table of contents, key concepts, original title, tests & measures, mesh word]

17	3 and 6 and 9 and 12 and 15 and 16

18	limit 17 to (english language and yr="2014 – 2026”)
