# Supplementary material for: Public knowledge, beliefs and behaviours related to antimicrobial resistance and antibiotics: a systematic review of studies in high-income countries
Source: JAC Antimicrob Resist. 2026 Sep 30;8(5):dlag213. doi: 10.1093/jacamr/dlag213 (PMC13624658; doi:10.1093/jacamr/dlag213)
Supplement: Supplementary file 1 [file dlag213_supplementary_data.zip › Supplementary file 2 - Coded direction of items.docx]

**Table 1.** Coding direction of items used to measure public knowledge, beliefs and behaviours related to antibiotics and antimicrobial resistance, split by outcome, domains, and sub-domains

| **Outcome** | **Domain** | **Sub-domain** | **Direction of coding** | **Item and direction of coding** |
| --- | --- | --- | --- | --- |
| Knowledge | Knowledge of AMR | Sub-domain 1. Causes of AMR | % said no/don’t know/false/disagree | 1.1 Overprescription of/easy access to antibiotics |
|  |  |  |  | 1.2 Overuse/use of antibiotics in livestock |
|  |  |  |  | 1.3 Absence of antibiotics being discovered |
|  |  |  |  | 1.4 Poor infection control in healthcare settings |
|  |  |  |  | 1.5 Poor hygiene and sanitation |
|  |  |  |  | 1.6 Travelling outside own country |
|  |  |  |  | 1.7 Lack of awareness/information |
|  |  |  |  | 1.8 Inadequate countermeasures |
|  |  |  |  | 1.9 Drug interactions |
|  |  |  |  | 1.10 Inappropriate use of antibiotics (Not finishing the course/Incorrect use/Overuse/Non-indicated use/Non-prescribed/Excessive use) |
|  |  | Sub-domain 2. Heard of terms | % said no/don’t know/false/disagree | 2.1 MDR pathogens |
|  |  |  |  | 2.2 Antibiotic resistance |
|  |  |  |  | 2.3 AMR |
|  |  |  |  | 2.4 Antimicrobial resistance |
|  |  |  |  | 2.5 Drug resistance |
|  |  |  |  | 2.6 Antibiotic-resistant bacteria |
|  |  |  |  | 2.7 Superbugs |
|  |  |  |  | 2.8 ESKAPE pathogens |
|  |  | Sub-domain 3. Consequences of AMR | % said no/don’t know/false/disagree | 3.1 AMR threatens medical procedures |
|  |  |  |  | 3.2 AMR is a threat to health |
|  |  |  | % said yes/don’t know/true/agree | 3.3 ABR is only an issue in hospitals |
|  |  |  |  | 3.4 ABR affects common infections |
|  |  |  |  | 3.5 I am not at risk of AMR if I use antibiotics appropriately |
|  |  |  |  | 3.6 How other people use antibiotics doesn't impact my chance of impact |
|  |  |  |  | 3.7 How I use antibiotics doesn't impact others' chance of impact |
|  |  |  |  | 3.8 Infections caused by resistant bacteria can be easily cured |
|  |  |  |  | 3.9 AMR is only an issue for people who use antibiotics regularly |
|  |  |  |  | 3.10 An antibiotic will remain effective against the same disease in the future |
|  |  | Sub-domain 4. Existence of AMR | % said no/don’t know/false/disagree | 4.1 Bacteria can become resistant to antibiotics |
|  |  |  |  | 4.2 AMR is the survival of bacteria against antibiotics |
|  |  |  |  | 4.3 Resistant bacteria can spread from animals to humans |
|  |  |  |  | 4.4 Resistant bacteria can spread from person to person |
|  |  |  | % said yes/don’t know/true/agree | 4.5 People can become resistant to antibiotics |
|  |  |  |  | 4.6 Viruses can become resistant to antibiotics |
|  |  |  | % said no/don’t know/false/disagree | 4.7 People can be a "carrier" of resistant bacteria |
|  |  |  |  | 4.8 Resistant bacteria is a problem in my country |
|  |  |  |  | 4.9 Infections by resistant bacteria are increasing |
|  |  |  | % said yes/don’t know/true/agree | 4.10 ABR occurs when antibiotics become less powerful |
|  |  |  | % said no/don’t know/false/disagree | 4.11 ABR can affect anyone |
|  |  |  |  | 4.12 AMR can impact me/my family |
|  |  |  | % said yes/don’t know/true/agree | 4.13 AMR occurs when your body becomes resistant to antibiotics |
|  |  |  | % said no/don’t know/false/disagree | 4.14 ESKAPE pathogens can resist practically all types of treatment used to fight against them |
|  |  |  | % said yes/don’t know/true/agree | 4.15 ESKAPE pathogens are only found in hospitals and healthcare settings |
|  |  |  |  | 4.16 ESKAPE pathogens are typically harmless and rarely cause infections in humans |
|  |  |  | % said no/don’t know/false/disagree | 4.17 ESKAPE pathogens are a major threat to public health globally |
|  |  | Sub-domain 5. Tackling AMR |  | 5.1 The antibiogram evaluates antibiotic sensitivity |
|  |  |  |  | 5.2 The public can help prevent antibiotic resistance |
|  |  |  |  | 5.3 I am aware of measures to prevent antibiotic resistance |
|  |  |  |  | 5.4 I am willing to learn more about AMR |
|  | Knowledge of antibiotics | Sub-domain 1. Appropriate antibiotic use | % said yes/don’t know/true/agree | 1.1 It is acceptable to share antibiotics |
|  |  |  |  | 1.2 It is acceptable to stop antibiotics before finishing the course |
|  |  |  | % said no/don’t know/false/disagree | 1.3 The effectiveness of treatment is reduced if a full course of AB is not completed |
|  |  |  | % said yes/don’t know/true/agree | 1.4 It is acceptable to use antibiotics without consulting a doctor |
|  |  |  |  | 1.5 It is better to give an antibiotic intravenously for any case |
|  |  |  | % said no/don’t know/false/disagree | 1.6 Unnecessary use of antibiotics can make them less effective |
|  |  |  | % said yes/don’t know/true/agree | 1.7 It is acceptable to buy/request the same antibiotics based on previous experience |
|  |  |  |  | 1.8 Antibiotics should not be taken without an antibiogram |
|  |  |  |  | 1.9 It is ok to keep and/or use leftover antibiotics |
|  |  |  |  | 1.10 It is okay to miss doses during the course of antibiotics |
|  |  |  |  | 1.11 Self-medication with antibiotics is good practice |
|  |  | Sub-domain 2. Antibiotic purposes and effects | % said no/don’t know/false/disagree | 2.1 Antibiotics are effective against bacteria |
|  |  |  | % said yes/don’t know/true/agree | 2.2 Antibiotics are effective against viruses |
|  |  |  |  | 2.3 Antibiotics are effective against cold/flu |
|  |  |  |  | 2.4 Antibiotics are effective against UTIs/baldder infections |
|  |  |  | % said no/don’t know/false/disagree | 2.5 Antibiotics can kill naturally occurring bacteria |
|  |  |  |  | 2.6 Antibiotics have side effects |
|  |  |  |  | 2.7 Antibiotics are useful for pain relief |
|  |  |  | % said yes/don’t know/true/agree | 2.8 Fascia A antibiotics are only used in hospitals |
|  |  |  |  | 2.9 Antibiotics can cure all infections |
|  |  |  |  | 2.10 Antibiotics are used to stop fever |
|  |  |  |  | 2.11 Antibiotics are the same as anti-inflammatory |
|  |  |  | % said no/don’t know/false/disagree | 2.12 Antibiotics are effective against skin infections |
|  |  |  |  | 2.13 I can get well from a bacterial infection without antibiotics |
|  |  |  |  | 2.14 Antibiotics can cause vaginal fungus infection in women |
|  |  |  |  | 2.15 Antibiotics may influence the effect of other medications |
|  |  |  |  | 2.16 Other medications may influence the effect of antibiotics |
|  |  |  |  | 2.17 Using antibiotics with alcohol can be dangerous |
|  |  |  | % said yes/don’t know/true/agree | 2.18 Expensive antibiotics are more effective than cheaper ones |
|  |  |  |  | 2.19 Higher doses of antibiotics result in faster recovery |
|  |  |  |  | 2.20 Antibiotics are effective against COVID-19 |
|  |  |  |  | 2.21 One antibiotic is as good as another |
|  |  |  |  | 2.22 Ear infections in children always need to be treated with antibiotics |
|  |  |  |  | 2.23 Antibiotics can prevent any illness from becoming worse |
|  |  |  |  | 2.24 Lower doses of antibiotics cause fewer side effects |
|  |  |  |  | 2.25 Antibiotics are effective against gonorrhoea |
|  |  |  |  | 2.26 Antibiotics are effective against HIV/AIDS |
|  |  |  |  | 2.27 Antibiotics are effective against headaches |
|  |  |  |  | 2.28 Antibiotics are effective against measles |
|  |  |  |  | 2.29 Antibiotics are effective against body aches |
|  |  |  |  | 2.30 Antibiotics are effective against malaria |
|  |  |  |  | 2.31 Antibiotics are effective against diarrhoea |
|  |  |  |  | 2.32 Antibiotics are effective against sore throat |
|  |  | Sub-domain 3. Appropriate antibiotic prescription | % said no/don’t know/false/disagree | 3.1 The sale of antibiotics without prescription is prohibited |
|  |  | Sub-domain 4. Identification of antibiotics |  | 4.1 Penicillin is an antibiotic |
|  |  |  | % said yes/don’t know/true/agree | 4.2 Paracetomol is an antibiotic |
|  |  |  | % said no/don’t know/false/disagree | 4.3 Tetracycline is an antibiotic |
|  |  |  | % said yes/don’t know/true/agree | 4.4 Ibuprofen is an antibiotic |
|  |  |  | % said no/don’t know/false/disagree | 4.5 Amoxicillin is an antibiotic |
| Beliefs | AMR risk perception | _ | % said no/don’t know/false/disagree | 1.1 Concerned about the amount of antibiotics used in animals |
|  |  |  |  | 1.2 Antibiotic resistance is already a global issue today |
|  |  |  |  | 1.3 Antibiotic resistance is already an issue in my country |
|  |  |  |  | 1.4 Antibiotic resistance can impact my families' and my own health |
|  |  |  | % said yes/don’t know/true/agree | 1.5 Antibiotic resistance is only an issue for people who take antibiotics regularly |
|  |  |  | % said no/don’t know/false/disagree | 1.6 Antibiotic resistance is a serious problem |
|  |  |  |  | 1.7 The topic of AMR is important |
|  |  |  | % said yes/don’t know/true/agree | 1.8 Antibiotics do not cause harm |
|  |  |  | % said no/don’t know/false/disagree | 1.9 I am concerned about the issue of antibiotic resistance |
|  | Attribution of responsibility |  |  | 2.1 Farmers are responsible for reducing the use of antibiotics in livestock breeding. |
|  |  |  |  | 2.2 Politicians are responsible for reducing the use of antibiotics in livestock breeding |
|  |  |  |  | 2.3 Consumers are responsible for reducing the use of antibiotics in livestock breeding |
|  |  |  |  | 2.4 Each individual has a responsibility to correctly take antibiotics to reduce the spread of AMR |
|  |  |  |  | 2.5 Healthcare staff are responsible for reducing/combatting the spread of AMR |
|  |  |  |  | 2.6 Politicians are responsible for reducing/combatting the spread of AMR |
|  |  |  |  | 2.7 The doctor should not give the patient an antibiotic when they think they do not need them |
|  |  |  | % said yes/don’t know/true/agree | 2.8 The doctor should give me an antibiotic when I think I need them |
|  |  |  |  | 2.8 The doctor who prescribes antibiotics for cold are good |
|  | Antibotic use |  |  | 3.1 I think natural sources (e.g., ginger, honey) may work better than antibiotics |
|  |  |  |  | 3.2 It is easy to obtain antibiotics without a prescription |
|  | Expectations and preferences |  |  | 4.1 Antibiotics should be accessed without a prescription |
|  |  |  |  | 4.2 When I go to the doctor with a cold or flu, I expect an antibiotic |
|  |  |  |  | 4.3 I prefer to have antibiotics in my cupboard at home for an emergency |
|  |  |  |  | 4.4 I prefer doctors who prescribe |
|  |  |  |  | 4.5 I prefer intravenous antibiotics |
|  |  |  |  | 4.6 I expect antibiotics when visiting a doctor |
|  | Trust |  | % said no/don’t know/false/disagree | 5.1 I trusted the physician's decision when they were deciding not to prescribe an antibiotic |
|  |  |  |  | 5.2 I trust that healthcare professionals are prescribing antibiotics appropriately |
|  | Self-efficacy |  |  | 6.1 Individual people can have an impact on drug resistant infections |
|  |  |  | % said yes/don’t know/true/agree | 6.2 I can successfully treat common infectious diseases with antibiotics on my own |
|  |  |  | % said no/don’t know/false/disagree | 6.3 Public education is needed to combat AMR |
|  | Response efficacy |  | % said yes/don’t know/true/agree | 7.1 Nothing much can be done to stop resistance |
|  |  |  |  | 7.2 Experts will solve resistance before it's serious |
| Behaviours | Antibiotic obtainment | Sub-domain 1. Positive behaviours | % said yes/don’t know/true/agree/always/often/sometimes | I only take antibiotics after medical consultation |
|  |  |  |  | Prescription is my source of antibiotic obtainment |
|  |  |  |  | I obtained antibiotics from a medical store or pharmacy |
|  |  | Sub-domain 2. Negative behaviours |  | I have asked the GP for antibiotics |
|  |  |  |  | I have used someone else's antibiotics |
|  |  |  |  | I have used antibiotics sourced from the Internet |
|  |  |  |  | I would change physician if they would not prescribe me an antibiotic |
|  |  |  |  | I obtained antibiotics without a prescription |
|  | Antibiotic use | Sub-domain 1. Positive behaviours |  | I would/have finished a course of antibiotics |
|  |  |  |  | I would take/have taken antibiotics as recommended by the HCP/instructions |
|  |  |  |  | I take antibiotics when I have a UTI |
|  |  |  |  | I read the drug's leaflet |
|  |  |  |  | I read/check the drug's expiration date |
|  |  |  |  | I would/have consulted a HCP if I took my antibiotics incorrectly |
|  |  |  |  | I store my antibiotics as directed |
|  |  |  |  | I stop taking antibiotics after they run out |
|  |  |  |  | I stop taking antibiotics after consulting a HCP |
|  |  |  |  | When I have problems occurring while taking antibiotics, I consult a HCP |
|  |  | Sub-domain 2. Negative behaviours |  | I have shared antibiotics with others |
|  |  |  |  | I have/had saved antibiotics at home to use if necessary |
|  |  |  |  | I would stop/have stopped taking antibiotics early/as soon as I feel better |
|  |  |  |  | I take antibiotics when I have the cold/flu |
|  |  |  |  | I take antibiotics to treat the same symptoms as before |
|  |  |  |  | I stop antibiotics if I forget to take a dose |
|  |  |  |  | I have taken antibiotics without a prescription |
|  |  |  |  | I have taken antibiotics that I used previously and it cured the illness |
|  |  |  |  | When self-medicating, I change antibiotics |
|  |  |  |  | When self-medicating, I change doses |
|  |  |  |  | I self-medicate with antibiotics |
|  |  |  |  | I stop using the antibiotics if I get expected side effects |
|  |  |  |  | I have used antibiotics without a known infection |
|  |  |  |  | I stop taking antibiotics after a few days regardless of the outcome |
|  |  |  |  | I stop taking antibiotics a few days after the recovery |
|  |  |  |  | I will take leftover antibiotics when I think I need them |
|  | Antibiotic disposal | Sub-domain 1. Positive behaviours |  | I would leave leftover antibiotics to a pharmacy |
|  |  |  |  | I would leave leftover antibiotics in the special waste |
|  |  | Sub-domain 2. Negative behaviours |  | I would leave leftover antibiotics in the domestic waste |
|  |  |  |  | I would flush leftover antibiotics |
|  | Information seeking | Sub-domain 1. Positive behaviours |  | I seek information about the correct use of antibiotics from reliable sources |
|  |  |  |  | I seek information from HCPs |
|  |  |  |  | I seek information from health education programs |
|  |  | Sub-domain 2. Negative behaviours |  | I seek information from family and friends |
