# Supplementary material for: Public knowledge, beliefs and behaviours related to antimicrobial resistance and antibiotics: a systematic review of studies in high-income countries
Source: JAC Antimicrob Resist. 2026 Sep 30;8(5):dlag213. doi: 10.1093/jacamr/dlag213 (PMC13624658; doi:10.1093/jacamr/dlag213)
Supplement: Supplementary file 1 [file dlag213_supplementary_data.zip › Supplementary file 3 - Country level results.docx]

**Table 2.** Country-level knowledge sub-domain results from a systematic review of studies in high-income countries of public knowledge, beliefs and behaviours related to antimicrobial resistance and antibiotics (k=33)

|  |  | Knowledge of AMR sub-domains | | | | | Knowledge of antibiotics sub-domains | | | |
| --- | --- | --- | --- | --- | --- | --- | --- | --- | --- | --- |
| Country | Values | Causes of AMR | Heard of terms | Consequences of AMR | Existence of AMR | Tackling AMR | Appropriate antibiotic use | Antibiotic purpose and effect | Identification of antibiotics | Appropriate antibiotic prescription |
| UK | Mean | 47.33 | - | - | - | - | 14.00 | - | - | - |
|  | Standard Deviation | - | - | - | - | - |  | - | - | - |
|  | Median | 47.33 | - | - | - | - | 14.00 | - | - | - |
|  | Minimum | 47.33 | - | - | - | - | 14.00 | - | - | - |
|  | Maximum | 47.33 | - | - | - | - | 14.00 | - | - | - |
|  | *k* | 1 | - | - | - | - | 1 | - | - | - |
| Germany | Mean | 12.74 | 5.10 | 28.00 | 62.00 |  | 29.50 | 37.80 | - | - |
|  | Standard Deviation | 12.39 | - | - | - | - | - | - | - | - |
|  | Median | 12.74 | 5.10 | 28.00 | 62.00 | - | 29.50 | 37.80 | - | - |
|  | Minimum | 3.98 | 5.10 | 28.00 | 62.00 | - | 29.50 | 37.80 | - | - |
|  | Maximum | 21.50 | 5.10 | 28.00 | 62.00 | - | 29.50 | 37.80 | - | - |
|  | *k* | 2 | 1.00 | 1.00 | 1.00 | - | 1 | 1.00 | - | - |
| Italy | Mean | 40.89 | - | - | 58.94 | 47.50 | 35.19 | 41.73 | - | 44.47 |
|  | Standard Deviation | - | - | - | - | - | - | - | - | - |
|  | Median | 40.89 | - | - | 58.94 | 47.50 | 35.19 | 41.73 | - | 44.47 |
|  | Minimum | 40.89 | - | - | 58.94 | 47.50 | 35.19 | 41.73 | - | 44.47 |
|  | Maximum | 40.89 | - | - | 58.94 | 47.50 | 35.19 | 41.73 | - | 44.47 |
|  | *k* | 1 | - | - | 1 | 1 | 1 | 1 | - | 1 |
| Sweden | Mean | 44.18 | - | - | 61.00 | - | 10.00 | 28.98 | - | - |
|  | Standard Deviation | - | - | - | - | - | - | - | - | - |
|  | Median | 44.18 | - | - | 61.00 | - | 10.00 | 28.98 | - | - |
|  | Minimum | 44.18 | - | - | 61.00 | - | 10.00 | 28.98 | - | - |
|  | Maximum | 44.18 | - | - | 61.00 | - | 10.00 | 28.98 | - | - |
|  | *k* | 1 | - | - | 1 | - | 1 | 1 | - | - |
| Saudi Arabia | Mean | 30.86 | 48.50 | 42.41 | 44.71 | 28.04 | 26.66 | 47.80 | - | - |
|  | Standard Deviation | 19.48 | 6.94 | 13.45 | 26.33 | 34.00 | 19.47 | 9.89 | - | - |
|  | Median | 24.10 | 48.50 | 42.24 | 40.20 | 28.04 | 17.29 | 50.60 | - | - |
|  | Minimum | 16.66 | 43.59 | 29.85 | 20.92 | 4.00 | 9.87 | 32.45 | - | - |
|  | Maximum | 58.57 | 53.40 | 55.30 | 73.00 | 52.08 | 61.03 | 58.56 | - | - |
|  | *k* | 4 | 2.00 | 4 | 3 | 2 | 6 | 7 | - | - |
| Greece | Mean | 46.25 | - | - | 55.64 | 38.90 | 9.60 | 30.89 | 37.02 | - |
|  | Standard Deviation |  | - | - | - | - | - | - | - | - |
|  | Median | 46.25 | - | - | 55.64 | 38.90 | 9.60 | 30.89 | 37.02 | - |
|  | Minimum | 46.25 | - | - | 55.64 | 38.90 | 9.60 | 30.89 | 37.02 | - |
|  | Maximum | 46.25 | - | - | 55.64 | 38.90 | 9.60 | 30.89 | 37.02 | - |
|  | *k* | 1 | - | - | 1 | 1 | 1 | 1 | 1 | - |
| Singapore | Mean | - | 54.46 | 52.58 | 52.38 | - | 19.37 | 47.23 | - | - |
|  | Standard Deviation | - | 13.85 | 0.59 | 8.36 | - | 1.24 | 4.33 | - | - |
|  | Median | - | 54.46 | 52.58 | 52.38 | - | 19.37 | 47.23 | - | - |
|  | Minimum | - | 44.67 | 52.16 | 46.47 | - | 18.50 | 44.17 | - | - |
|  | Maximum | - | 64.25 | 53.00 | 58.29 | - | 20.25 | 50.29 | - | - |
|  | *k* | - | 2 | 2 | 2 | - | 2 | 2 | - | - |
| UAE | Mean | - | - | - | - | - | 34.18 | - | - | - |
|  | Standard Deviation | - | - | - | - | - | - | - | - | - |
|  | Median | - | - | - | - | - | 34.18 | - | - | - |
|  | Minimum | - | - | - | - | - | 34.18 | - | - | - |
|  | Maximum | - | - | - | - | - | 34.18 | - | - | - |
|  | *k* | - | - | - | - | - | 1 | - | - | - |
| Cyprus | Mean | - | 34.00 | - | 90.00 | - | - | 25.57 | - | - |
|  | Standard Deviation | - |  | - |  | - | - |  | - | - |
|  | Median | - | 34.00 | - | 90.00 | - | - | 25.57 | - | - |
|  | Minimum | - | 34.00 | - | 90.00 | - | - | 25.57 | - | - |
|  | Maximum | - | 34.00 | - | 90.00 | - | - | 25.57 | - | - |
|  | *k* | - | 1 | - | 1 | - | - | 1 | - | - |
| Japan | Mean | 80.70 | 58.40 | - | 73.45 | - | 32.50 | 71.57 | - | - |
|  | Standard Deviation | - | - | - | - | - | - | - | - | - |
|  | Median | 80.70 | 58.40 | - | 73.45 | - | 32.50 | 71.57 | - | - |
|  | Minimum | 80.70 | 58.40 | - | 73.45 | - | 32.50 | 71.57 | - | - |
|  | Maximum | 80.70 | 58.40 | - | 73.45 | - | 32.50 | 71.57 | - | - |
|  | *k* | 1 | 1 | - | 1 | - | 1 | 1 | - | - |
| France | Mean | 53.00 | - | 25.00 | - | - |  |  | - | - |
|  | Standard Deviation | - | - | - | - | - | - | - | - | - |
|  | Median | 53.00 | - | 25.00 | - | - | - | - | - | - |
|  | Minimum | 53.00 | - | 25.00 | - | - | - | - | - | - |
|  | Maximum | 53.00 | - | 25.00 | - | - | - | - | - | - |
|  | *k* | 1 | - | 1 | - | - | - | - | - | - |
| Romania | Mean | - | - | - | - | - | - | 93.00 | - | - |
|  | Standard Deviation | - | - | - | - | - | - |  | - | - |
|  | Median | - | - | - | - | - | - | 93.00 | - | - |
|  | Minimum | - | - | - | - | - | - | 93.00 | - | - |
|  | Maximum | - | - | - | - | - | - | 93.00 | - | - |
|  | *k* | - | - | - | - | - | - | 1 | - | - |
| Oman | Mean | 50.30 | - | 32.30 | - | - | 23.90 | 52.90 | - | - |
|  | Standard Deviation | - | - | - | - | - | - | - | - | - |
|  | Median | 50.30 | - | 32.30 | - | - | 23.90 | 52.90 | - | - |
|  | Minimum | 50.30 | - | 32.30 | - | - | 23.90 | 52.90 | - | - |
|  | Maximum | 50.30 | - | 32.30 | - | - | 23.90 | 52.90 | - | - |
|  | *k* | 1 | - | 1 | - | - | 1 | 1 | - | - |
| Ireland | Mean | - | 48.73 | 24.97 | 36.70 | - | 26.63 | 33.56 | - | - |
|  | Standard Deviation | - | - | - | - | - | - | - | - | - |
|  | Median | - | 48.73 | 24.97 | 36.70 | - | 26.63 | 33.56 | - | - |
|  | Minimum | - | 48.73 | 24.97 | 36.70 | - | 26.63 | 33.56 | - | - |
|  | Maximum | - | 48.73 | 24.97 | 36.70 | - | 26.63 | 33.56 | - | - |
|  | *k* | - | 1 | 1 | 1 | - | 1 | 1 | - | - |
| Northern Ireland | Mean | - | 44.69 | 20.80 | 33.77 | - | 20.80 | 27.58 | - | - |
|  | Standard Deviation | - | - | - | - | - | - | - | - | - |
|  | Median | - | 44.69 | 20.80 | 33.77 | - | 20.80 | 27.58 | - | - |
|  | Minimum | - | 44.69 | 20.80 | 33.77 | - | 20.80 | 27.58 | - | - |
|  | Maximum | - | 44.69 | 20.80 | 33.77 | - | 20.80 | 27.58 | - | - |
|  | *k* | - | 1 | 1 | 1 | - | 1 | 1 | - | - |

**Note.** Only countries which contributed to the knowledge outcome are presented.

**Table 3.** Country-level belief domain results from a systematic review of studies in high-income countries of public knowledge, beliefs and behaviours related to antimicrobial resistance and antibiotics (k=33)

| **Country** | **Values** | **AMR risk perception** | **Attribution of responsibility** | **Antibiotic use** | **Expectations and preferences** | **Trust** | **Self-efficacy** | **Response efficacy** |
| --- | --- | --- | --- | --- | --- | --- | --- | --- |
| UK | Mean | 47.13 | - | - | - | - | 13.40 | - |
|  | Standard Deviation | - | - | - | - | - | - | - |
|  | Median | 47.13 | - | - | - | - | 13.40 | - |
|  | Minimum | 47.13 | - | - | - | - | 13.40 | - |
|  | Maximum | 47.13 | - | - | - | - | 13.40 | - |
|  | *k* | 1 | - | - | - | - | 1 | - |
| Germany | Mean | 29.28 | 35.77 | - | 24.50 | - | - | - |
|  | Standard Deviation | 13.60 | - | - | - | - | - | - |
|  | Median | 29.28 | 35.77 | - | 24.50 | - | - | - |
|  | Minimum | 19.66 | 35.77 | - | 24.50 | - | - | - |
|  | Maximum | 38.9 | 35.77 | - | 24.50 | - | - | - |
|  | *k* | 2 | 1 | - | 1 | - | - | - |
| Italy | Mean | 23.23 | - | - | - | - | - | - |
|  | Standard Deviation | - | - | - | - | - | - | - |
|  | Median | 23.23 | - | - | - | - | - | - |
|  | Minimum | 23.23 | - | - | - | - | - | - |
|  | Maximum | 23.23 | - | - | - | - | - | - |
|  | *k* | 1 | - | - | - | - | - | - |
| Saudi Arabia | Mean | 43.49 | 13.37 | 36.30 | 32.99 | 27.49 | 34 | - |
|  | Standard Deviation | 11.68 | 4.34 |  | 34.39 | 35.96 | - | - |
|  | Median | 43.49 | 13.37 | 36.30 | 32.99 | 7.45 | 34 | - |
|  | Minimum | 35.23 | 10.30 | 36.30 | 8.67 | 6.02 | 34 | - |
|  | Maximum | 51.75 | 16.44 | 36.30 | 57.30 | 69 | 34 | - |
|  | *k* | 2 | 2 | 1 | 2 | 3 | 1 | - |
| Greece | Mean | - | - | 52.60 | - | - | - | - |
|  | Standard Deviation | - | - |  | - | - | - | - |
|  | Median | - | - | 52.60 | - | - | - | - |
|  | Minimum | - | - | 52.60 | - | - | - | - |
|  | Maximum | - | - | 52.60 | - | - | - | - |
|  | *k* | - | - | 1 | - | - | - | - |
| Singapore | Mean | - | - | - | 21.20 | - | - | - |
|  | Standard Deviation | - | - | - | 3.11 | - | - | - |
|  | Median | - | - | - | 21.20 | - | - | - |
|  | Minimum | - | - | - | 19 | - | - | - |
|  | Maximum | - | - | - | 23.40 | - | - | - |
|  | *k* | - | - | - | 2 | - | - | - |
| Cyprus | Mean | 64.10 | - | - | - | - | - | 72.20 |
|  | Standard Deviation | - | - | - | - | - | - | - |
|  | Median | 64.10 | - | - | - | - | - | 72.20 |
|  | Minimum | 64.10 | - | - | - | - | - | 72.20 |
|  | Maximum | 64.10 | - | - | - | - | - | 72.20 |
|  | *k* | 1 | - | - | - | - | - | 1 |
| Japan | Mean | - | 25.10 | - | 30.25 | - | - | - |
|  | Standard Deviation | - | - | - | - | - | - | - |
|  | Median | - | 25.10 | - | 30.25 | - | - | - |
|  | Minimum | - | 25.10 | - | 30.25 | - | - | - |
|  | Maximum | - | 25.10 | - | 30.25 | - | - | - |
|  | *k* | - | 1 | - | 1 | - | - | - |
| Oman | Mean | - | - | - | - | - | 9.70 | - |
|  | Standard Deviation | - | - | - | - | - | - | - |
|  | Median | - | - | - | - | - | 9.70 | - |
|  | Minimum | - | - | - | - | - | 9.70 | - |
|  | Maximum | - | - | - | - | - | 9.70 | - |
|  | *k* | - | - | - | - | - | 1 | - |

**Note.** Only countries which contributed to the belief outcome are presented.

**Table 4.** Country-level behaviour sub-domain results from a systematic review of studies in high-income countries of public knowledge, beliefs and behaviours related to antimicrobial resistance and antibiotics (k=33)

| **Country** | **Values** | **Positive antibiotic obtainment** | **Negative antibiotic obtainment** | **Positive antibiotic use** | **Negative antibiotic use** | **Positive antibiotic disposal** | **Negative antibiotic disposal** | **Positive information seeking** | **Negative information seeking** |
| --- | --- | --- | --- | --- | --- | --- | --- | --- | --- |
| UK | Mean | - | - | 91.00 | - | - | - | - | - |
|  | Standard Deviation | - | - | - | - | - | - | - | - |
|  | Median | - | - | 91.00 | - | - | - | - | - |
|  | Minimum | - | - | 91.00 | - | - | - | - | - |
|  | Maximum | - | - | 91.00 | - | - | - | - | - |
|  | *k* | - | - | 1 | - | - | - | - | - |
| Germany | Mean | - | 6.30 | 70.68 | 9.28 | 15.50 | 23.00 | - | - |
|  | Standard Deviation | - | - | 35.38 | 7.14 | - | - | - | - |
|  | Median | - | 6.30 | 70.68 | 9.28 | 15.50 | 23.00 | - | - |
|  | Minimum | - | 6.30 | 45.66 | 4.23 | 15.50 | 23.00 | - | - |
|  | Maximum | - | 6.30 | 95.70 | 14.33 | 15.50 | 23.00 | - | - |
|  | *k* | - | 1 | 2 | 2 | 1 | 1 | - | - |
| Italy | Mean | 99.48 | - | 83.36 | 59.28 | - | - | - | - |
|  | Standard Deviation | - | - | - | - | - | - | - | - |
|  | Median | 99.48 | - | 83.36 | 59.28 | - | - | - | - |
|  | Minimum | 99.48 | - | 83.36 | 59.28 | - | - | - | - |
|  | Maximum | 99.48 | - | 83.36 | 59.28 | - | - | - | - |
|  | *k* | 1 | - | 1 | 1 | - | - | - | - |
| USA | Mean | 23.00 | 47.00 | - | 18.00 | - | - | - | - |
|  | Standard Deviation | - | - | - | - | - | - | - | - |
|  | Median | 23.00 | 47.00 | - | 18.00 | - | - | - | - |
|  | Minimum | 23.00 | 47.00 | - | 18.00 | - | - | - | - |
|  | Maximum | 23.00 | 47.00 | - | 18.00 | - | - | - | - |
|  | *k* | 1 | 1 | - | 1 | - | - | - | - |
| Saudi Arabia | Mean | 70.56 | 28.60 | 63.34 | 40.88 | 0.70 | 25.10 | 70.61 | 29.80 |
|  | Standard Deviation | 16.43 | 21.65 | 25.06 | 16.62 | - | - | 34.87 | - |
|  | Median | 74.00 | 27.35 | 58.70 | 39.41 | 0.70 | 25.10 | 70.61 | 29.80 |
|  | Minimum | 52.69 | 6.23 | 35.00 | 15.92 | 0.70 | 25.10 | 45.95 | 29.80 |
|  | Maximum | 85.00 | 63.60 | 94.40 | 64.00 | 0.70 | 25.10 | 95.26 | 29.80 |
|  | *k* | 3 | 6 | 7 | 10 | 1 | 1 | 2 | 1 |
| Greece | Mean |  | 11.00 | 93.50 | - | - | - | - | - |
|  | Standard Deviation | - | - | - | - | - | - | - | - |
|  | Median | - | 11.00 | 93.50 | - | - | - | - | - |
|  | Minimum | - | 11.00 | 93.50 | - | - | - | - | - |
|  | Maximum | - | 11.00 | 93.50 | - | - | - | - | - |
|  | *k* | - | 1 | 1 | - | - | - | - | - |
| Singapore | Mean | - | 9.20 | 74.15 | 8.78 | - | - | 31.66 | 27.00 |
|  | Standard Deviation | - | 3.11 | 18.88 | 10.22 | - | - |  |  |
|  | Median | - | 9.20 | 74.15 | 8.78 | - | - | 31.66 | 27.00 |
|  | Minimum | - | 7.00 | 60.80 | 1.55 | - | - | 31.66 | 27.00 |
|  | Maximum | - | 11.40 | 87.50 | 16.00 | - | - | 31.66 | 27.00 |
|  | *k* | - | 2 | 2 | 2 | - | - | 1 | 1 |
| UAE | Mean | - | - | 26.44 | 17.51 | 10.61 | 39.71 | - | - |
|  | Standard Deviation | - | - | - | - | - | - | - | - |
|  | Median | - | - | 26.44 | 17.51 | 10.61 | 39.71 | - | - |
|  | Minimum | - | - | 26.44 | 17.51 | 10.61 | 39.71 | - | - |
|  | Maximum | - | - | 26.44 | 17.51 | 10.61 | 39.71 | - | - |
|  | *k* | - | - | 1 | 1 | 1 | 1 | - | - |
| Cyprus | Mean | 75.60 | 38.65 | 87.60 | - | - | - | - | - |
|  | Standard Deviation | - | - | - | - | - | - | - | - |
|  | Median | 75.60 | 38.65 | 87.60 | - | - | - | - | - |
|  | Minimum | 75.60 | 38.65 | 87.60 | - | - | - | - | - |
|  | Maximum | 75.60 | 38.65 | 87.60 | - | - | - | - | - |
|  | *k* | 1 | 1 | 1 | - | - | - | - | - |
| Japan | Mean | - | 10.20 | - | - | - | - | 19.10 | - |
|  | Standard Deviation | - | - | - | - | - | - | - | - |
|  | Median | - | 10.20 | - | - | - | - | 19.10 | - |
|  | Minimum | - | 10.20 | - | - | - | - | 19.10 | - |
|  | Maximum | - | 10.20 | - | - | - | - | 19.10 | - |
|  | *k* | - | 1 | - | - | - | - | 1 | - |
| France | Mean | - | - | - | 22.00 | 64.00 | - | - | - |
|  | Standard Deviation | - | - | - |  |  | - | - | - |
|  | Median | - | - | - | 22.00 | 64.00 | - | - | - |
|  | Minimum | - | - | - | 22.00 | 64.00 | - | - | - |
|  | Maximum | - | - | - | 22.00 | 64.00 | - | - | - |
|  | *k* | - | - | - | 1 | 1 | - | - | - |
| Romania | Mean | - | - | 45.00 | 20.00 | - | - | - | - |
|  | Standard Deviation | - | - |  |  | - | - | - | - |
|  | Median | - | - | 45.00 | 20.00 | - | - | - | - |
|  | Minimum | - | - | 45.00 | 20.00 | - | - | - | - |
|  | Maximum | - | - | 45.00 | 20.00 | - | - | - | - |
|  | *k* | - | - | 1 | 1 | - | - | - | - |
| Oman | Mean | 36.80 | - | - | 49.40 | - | - | - | - |
|  | Standard Deviation |  | - | - |  | - | - | - | - |
|  | Median | 36.80 | - | - | 49.40 | - | - | - | - |
|  | Minimum | 36.80 | - | - | 49.40 | - | - | - | - |
|  | Maximum | 36.80 | - | - | 49.40 | - | - | - | - |
|  | *k* | 1 | - | - | 1 | - | - | - | - |
| Ireland | Mean | 91.85 | 2.35 | - | 0.30 | - | - | - | - |
|  | Standard Deviation | - | - | - | - | - | - | - | - |
|  | Median | 91.85 | 2.35 | - | 0.30 | - | - | - | - |
|  | Minimum | 91.85 | 2.35 | - | 0.30 | - | - | - | - |
|  | Maximum | 91.85 | 2.35 | - | 0.30 | - | - | - | - |
|  | *k* | 1 | 1 | - | 1 | - | - | - | - |
| Northern Ireland | Mean | 93.60 | 0.95 | - | 0.70 | - | - | - | - |
|  | Standard Deviation | - | - | - | - | - | - | - | - |
|  | Median | 93.60 | 0.95 | - | 0.70 | - | - | - | - |
|  | Minimum | 93.60 | 0.95 | - | 0.70 | - | - | - | - |
|  | Maximum | 93.60 | 0.95 | - | 0.70 | - | - | - | - |
|  | *k* | 1 | 1 | - | 1 | - | - | - | - |

**Note.** Only countries which contributed to the behaviour outcome are presented.
