# Supplementary material for: Public knowledge, beliefs and behaviours related to antimicrobial resistance and antibiotics: a systematic review of studies in high-income countries
Source: JAC Antimicrob Resist. 2026 Sep 30;8(5):dlag213. doi: 10.1093/jacamr/dlag213 (PMC13624658; doi:10.1093/jacamr/dlag213)
Supplement: Supplementary file 1 [file dlag213_supplementary_data.zip › Supplementary file 4 - MMAT criterion per study.docx]

**Table 1.** MMAT appraisal tool ratings for quantitative studies included in a systematic review of public knowledge, beliefs and behaviours related to antimicrobial resistance and antibiotics in high-income countries (k=29)

|  | Screening questions | | Quantitative descriptive MMAT questions | | | | |
| --- | --- | --- | --- | --- | --- | --- | --- |
|  | S1. Are there clear research questions? | S2. Do the collected data allow to address the research questions? | 4.1. Is the sampling strategy relevant to address the research question? | 4.2. Is the sample representative of the target population? | 4.3. Are the measurements appropriate? | 4.4. Is the risk of nonresponse bias low? | 4.5. Is the statistical analysis appropriate to answer the research question? |
| Smith & Buchan (2023) | Yes | Yes | Yes | Yes | Yes | Yes | Yes |
| Raupach-Rosin et al., (2019) | Yes | Yes | Yes | Yes | Yes | No | Yes |
| Schmiege et al., (2022) | Yes | Yes | Yes | Yes | Yes | yes | yes |
| Pennino (2023) | Yes | Yes | Yes | Yes | Yes | No | Yes |
| Adam & Bruce (2023) | Yes | No | Yes | No | Yes | Can't tell | Can't tell |
| Vallin et al., (2016) | Yes | Yes | Yes | Yes | Yes | Yes | Yes |
| Worthington et al., (2020) | Yes | Yes | Yes | Can't tell | Can't tell | Yes | Yes |
| Alshelwi (2018) | Yes | Yes | Yes | No | Can't tell | Can't tell | Yes |
| Alnasser et al., (2021) | Yes | Yes | Yes | No | Yes | Can't tell | Yes |
| Anderson (2022) | Yes | Yes | Yes | No | Yes | Can't tell | Yes |
| Bdair et al., (2024) | Yes | Yes | Yes | Yes | Yes | Yes | Yes |
| Benmerzouga et al., (2019) | Yes | Yes | Yes | Yes | No | Can't tell | Yes |
| Bhat et al., (2023) | Yes | Yes | No | Can't tell | Yes | No | Yes |
| Albadrani et al., (2023) | Yes | Yes | Yes | Yes | Yes | Yes | Yes |
| Al Saleh et al., (2021) | Yes | Yes | Yes | Yes | Yes | Yes | Yes |
| McCracken et al., (2023) | Yes | Yes | Yes | No | Yes | Can't tell | Yes |
| Lajunen et al., (2023) | Yes | Yes | Yes | No | Yes | No | Yes |
| Lee et al., (2023) | Yes | Yes | Yes | No | Yes | No | Yes |
| Lim et al., (2021) | Yes | Yes | Yes | Yes | Yes | Yes | Yes |
| Alhur et al., (2024) | Yes | Yes | Yes | Yes | Yes | Yes | Yes |
| Kharaba et al., (2024) | Yes | Yes | Yes | Yes | Yes | No | Can't tell |
| Ilktac et al., (2020) | Yes | Can't tell | Yes | Yes | Can't tell | No | Yes |
| Kamata et al., (2018) | Yes | Yes | Yes | Yes | Yes | Yes | Yes |
| Guo et al., (2021) | Yes | Yes | Yes | Yes | Yes | No | Yes |
| David et al., (2022) | Yes | Yes | Can't tell | Yes | Yes | Can't tell | Yes |
| El Zowalaty et al., (2016) | Yes | Yes | No | No | Yes | Yes | Yes |
| Boboia et al., (2020) | Can't tell | Can't tell | Yes | Can't tell | Can't tell | No | Can't tell |
| Osman et al., (2025) | Yes | Yes | Yes | No | Yes | Yes | Yes |
| Shields et al., (2026) | Yes | Yes | Yes | No | Yes | Yes | Yes |

**Table 2.** MMAT appraisal tool ratings for qualitative studies included in a systematic review of public knowledge, beliefs and behaviours related to antimicrobial resistance and antibiotics in high-income countries (k=4)

|  | Screening questions | | Qualitative MMAT questions | | | | |
| --- | --- | --- | --- | --- | --- | --- | --- |
|  | S1. Are there clear research questions? | S2. Do the collected data allow to address the research questions? | 1.1. Is the qualitative approach appropriate to answer the research question? | 1.2. Are the qualitative data collection methods adequate to address the research question? | 1.3. Are the findings adequately derived from the data? | 1.4. Is the interpretation of results sufficiently substantiated by data? | 1.5. Is there coherence between qualitative data sources, collection, analysis and interpretation? |
| Papadimou et al., (2022) | Yes | Yes | Yes | Yes | Yes | Yes | Yes |
| Lescure et al., (2022) | Yes | Yes | Yes | Yes | Yes | Yes | Yes |
| Davis et al., (2023) [57] | Yes | Yes | Yes | Yes | Yes | Yes | Yes |
| Ancillotti et al., (2018) | Yes | Yes | Yes | Yes | Yes | Yes | Yes |
